# Explainable AI in hospital clinical decision support systems: A scoping review of healthcare professionals’ perspectives

**DOI:** 10.1371/journal.pdig.0001417

**Published:** 2026-05-14

**Authors:** Bethany A. Van Dort, Thomas Engelsma, Pamela Sneekes, Linda Peute, Stephanie Medlock

**Affiliations:** 1 eHealth Living & Learning Lab Amsterdam, Department of Medical Informatics, Amsterdam UMC - University of Amsterdam, Amsterdam, the Netherlands; 2 Amsterdam Public Health Research Institute, Amsterdam UMC, Amsterdam, the Netherlands; 3 Department of Medical Informatics, Amsterdam UMC - University of Amsterdam, Amsterdam, the Netherlands; University of Bradford, UNITED KINGDOM OF GREAT BRITAIN AND NORTHERN IRELAND

## Abstract

Explainable Artificial Intelligence (XAI) has the potential to enhance clinical decision support (CDS) systems however, it remains unclear how XAI systems are perceived by healthcare professionals in hospital settings, and if new challenges arise as a result of explanations. This scoping review aimed to understand healthcare professionals’ perceptions of CDS systems with XAI in the hospital setting; specifically, the drivers of acceptance and use, explainability needs, and design preferences. Databases were searched. Studies were included if they reported qualitative findings on health professionals’ perceptions of XAI-enabled CDS systems used in hospital settings. MEDLINE, Embase, and Web of Science were searched, and reference lists were screened for additional papers. Study characteristics and health professional perceptions were extracted and inductively coded. A quality assessment was performed using the CASP checklist. Sixteen studies were identified. Included studies primarily focused on ML-based CDS systems for predicting various clinical outcomes. Most studies used feature importance or model agnostic techniques like SHapley Additive exPlanations (SHAP). Overall, healthcare professionals perceived CDS systems with XAI as useful for supporting clinical tasks, decision-making, and teamwork. Acceptance was influenced by integration into workflows, performance, data quality, and alignment with clinical knowledge. Concerns were raised about overreliance and reduced professional autonomy. Health professionals predominantly used explanations to validate outputs, and desired actionable information from systems. SHAP plots and visualizations were difficult to interpret. Participants preferred explanation designs that included concise, high-level information, and simple plots for quick interpretation. Clear visual indicators such as colour coding, contextual patient data, and aggregating similar features also aided interpretation. Poorly designed XAI explanations can hinder understanding and increase cognitive burden in busy clinical settings. Future research should optimise the design and delivery of explanations, so clinicians can appropriately trust in XAI CDS systems and feel confident in their clinical decision making.

## Author summary

In this review, we set out to understand how healthcare professionals feel about using clinical decision support systems that include explainable artificial intelligence (XAI). While these systems can make medical decision-making more transparent, it is not always clear how doctors and nurses actually experience them in real hospital settings. We reviewed sixteen studies that explored healthcare professionals’ opinions and experiences. Overall, most healthcare workers saw XAI-based tools as helpful for improving accuracy, efficiency, and collaboration in care. However, their acceptance depended on how well the systems fit into daily workflows, the quality of the data, and how trustworthy the outputs seemed. Explanations were mainly used to check if the system’s recommendations made sense, but many found the technical visualizations, like SHAP plots, confusing or time-consuming. Clinicians preferred simple, visual summaries with clear and relevant patient information. At the same time, some worried about becoming too reliant on AI or losing clinical independence. Our findings highlight that for XAI tools to truly support clinical practice, explanations must be designed to be quick, clear, and aligned with how clinicians think and work.

## Introduction

Artificial intelligence (AI) refers to the simulation of human intelligence in machines that are programmed to think, learn, and perform tasks that typically require human intelligence, such as problem-solving, decision-making, and pattern recognition [1]. Previous reviews of AI in healthcare have found AI significantly enhanced accuracy and efficiency in medical processes, particularly in diagnosis and treatment, potentially improving patient safety and reducing healthcare costs. [2,3] AI-powered tools, such as clinical decision support (CDS) systems, have shown promise in assisting healthcare professionals by providing timely, data-driven recommendations at the point of care. Despite this, concerns related to privacy, trust, transparency, and potential decision errors impacting patient safety have been raised by health professionals, hindering their acceptance and adoption in practice [2,3]. A previous scoping review found that 81% of studies reported healthcare professionals had concerns about AI systems, particularly regarding patient safety [3].

Explainable Artificial Intelligence (XAI) aims to enhance transparency, trustworthiness, and accountability of AI systems by providing explanations for their decisions [4,5]. While definitions of XAI vary in the literature, the term generally refers to two main approaches. One involves using inherently interpretable models, such as decision trees, and the other focuses on applying explainability techniques to complex ‘black box’ models to help users understand the model’s process or output [6,7]. This review will focus on the latter, due to the increasing complexity and opacity of machine learning (ML) models in healthcare [8]. Explainability techniques include model-agnostic methods, which can be applied to any model, or model-specific techniques, tailored to particular types of models. A central concept in many XAI methods is feature importance, which refers to the contribution of individual input variables ‘features’ to a model’s prediction, indicating how strongly each feature influences the output. For example, SHapley Additive exPlanations (SHAP) is a model-agnostic method used to quantify feature importance based on cooperative game theory, assigning each feature a contribution value that reflects its impact on a specific prediction [9]. Another commonly used method is Local Interpretable Model-agnostic Explanations (LIME), which approximates the predictions of a complex model locally using a simpler, interpretable model to explain individual predictions [9]. See Salih et al. [9] for visual examples of SHAP and LIME. Gradient-weighted Class Activation Mapping (GradCAM) is a model-specific technique providing visual explanations using heatmaps to highlight regions of interest, such as in radiological images [10]. SHAP was identified as the most frequently used XAI technique in healthcare, followed by Grad-CAM [11].

Previous reviews have explored XAI in healthcare, with a focus on explainability techniques [11,12], human computer interaction [13], and trust and intention to use systems [14]. Previous reviews of XAI have also reported that explainability is sensitive to contextual factors and future research should investigate different groups of end users [15,16]. Prior work has not focused on the needs and experiences of hospital healthcare professionals. Hospital settings are a particularly unique context, characterised by complex organisational structures, multidisciplinary teams, and high-pressure, fast-paced work [17,18]. These factors make hospitals a challenging setting for XAI implementation, particularly for CDS systems, which must deliver accurate predictions alongside explanations that are clear and useful for health professionals.

As previously mentioned, prior reviews of AI have found trust and transparency can impact AI adoption in healthcare [2,3]. While the addition of explainability aims to reduce these barriers, it remains unclear whether systems incorporating XAI are perceived as trustworthy and effective by healthcare professionals, or if new challenges arise as a result of providing explanations. By examining healthcare professionals’ views on XAI systems in hospitals, we can determine whether the challenges identified in previous AI studies persist, and whether new concerns emerge that are specific to XAI and the explainability techniques used. Additionally, design aspects, such as how explanations are presented and the clarity of visualizations, are important for interpretability. Understanding user perceptions of these design elements is important, as they directly affect whether explanations are interpretable and actionable in clinical practice.

Therefore, the aim of this scoping review was to understand healthcare professionals’ perceptions of CDS systems with XAI in the hospital setting; specifically, the drivers of acceptance and use, explainability needs, and design preferences.

## Materials and methods

A scoping review was conducted as XAI is a new field, and this method allowed for a broad mapping of existing literature and identification of key concepts related to health professionals’ perceptions of XAI in hospital settings [19]. The PRISMA-ScR (Preferred Reporting Items for Systematic reviews and Meta-Analyses extension for Scoping Reviews) guidelines [20] were followed in the reporting of this review and the checklist is included in the Supporting Information (S1 PRISMA Checklist). A review protocol was not pre-registered.

### Eligibility criteria

Papers were eligible if they were empirical research studies containing qualitative results on health professionals’ perceptions of CDS systems in a hospital setting that incorporated XAI. This includes health professionals’ thoughts, opinions, attitudes, or experiences using the XAI-system during prototype phases to post-implementation.

Reviews, commentaries and conference abstracts, and studies not in English were excluded. Studies conducted in community or primary care settings were excluded, as our focus was on hospital facilities. Studies that exclusively examined perceptions prior to system development were also excluded to ensure that perceptions were informed by actual system use.

### Search strategy

Online databases MEDLINE, Embase and Web of Science were searched. Keywords and database subject headings were defined and combined relating to (1) Explainable artificial intelligence (2) Hospital setting and (3) Perspective. See Supporting information (Table A in S1 Appendix) for the full search strategy. Searches were performed from database inception to the 15^th^ of June 2024. Reference lists of relevant systematic reviews and included papers were also searched for eligible papers.

### Study selection

Covidence [21] was used for screening. Papers were imported and the duplicates were removed by Covidence. Titles and abstracts of papers were screened independently by two researchers (BV and TE) and disagreements resolved by consensus. Full texts of included papers were then screened independently by two researchers (BV and TE). Disagreements were discussed with a third researcher (SM) until consensus was reached on included studies.

### Data extraction and synthesis

Data from each paper was extracted by at least two researchers independently to ensure accuracy (BV, PS, and TE). This included study characteristics, results relating to user perceptions of the system and specific design characteristics. The stage of development and implementation was also extracted and classified as ‘prototype’ if still in the early testing phase, ‘pre-implementation’ if the prototype is nearly finalised, ‘near-live’ if in the final stage of testing, and ‘post-implementation’ if conducting evaluations after implementation. Inductive coding was used to analyse and synthesise user perceptions. This involved researchers (BV, PS, and TE) independently coding results from four studies, and meeting to discuss and reach consensus on the themes and subthemes. Once themes and subthemes were clearly defined they were then used as a framework to analyse the remaining papers. New codes, when required, were discussed amongst researchers and added iteratively. Researchers (BV, PS and TE) then organised themes into (1) the CDS system with XAI in general, (2) the XAI techniques, and (3) design and usability.

### Quality assessment

A quality assessment was conducted using the Critical Appraisal Skills Programme (CASP) Checklist for qualitative studies [22]. Included papers were reviewed independently by at least two researchers (BV, PS and TE). For each of the CASP Checklist statements papers were assigned a Yes, No or Can’t tell. Researchers then met to discuss results and reach agreement on the classifications.

## Results

**Fig 1:**
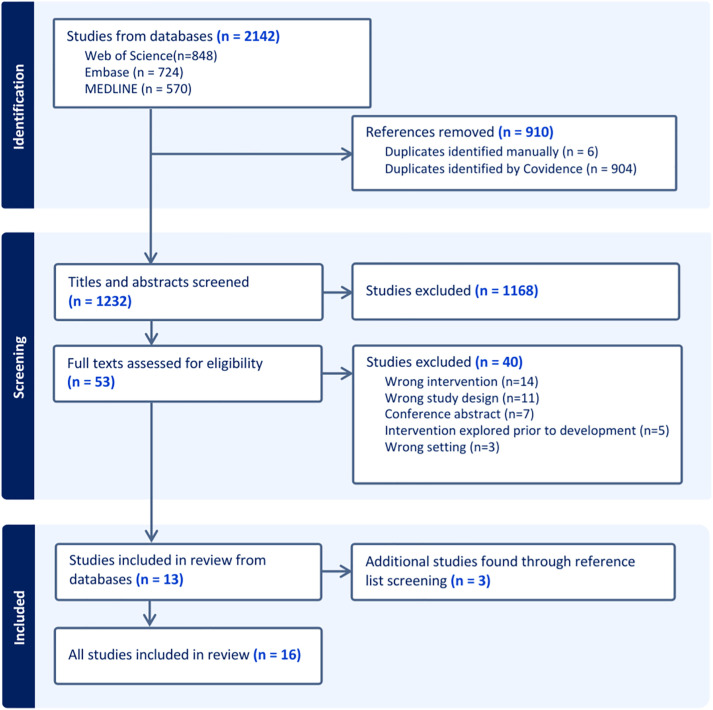
PRISMA flow diagram of the study selection process.

The search strategy retrieved 1232 papers from the databases after duplicates were removed. After screening, thirteen papers met the inclusion criteria and three papers were found from searching reference lists (see Fig 1).

### Study characteristics

Study characteristics are outlined in Table 1. The included studies were published from 2020 to 2024 and conducted in North America (n = 8), Europe (n = 7), and China (n = 1). Most focused on ML-based decision support tools aimed at predicting patient risks (see Table 1). While several systems had been implemented or were near live [26,30,31,33,36], the majority remained at prototype or pre-implementation stages. Integration with electronic health records (EHRs) was common, particularly in more mature tools [23,24,26–34,36]. Most studies used feature importance [26,29–32,34–36] and/or model agnostic techniques such as SHAP [23–25,27,28,34,38] and LIME [33,34,37]. Four studies used more than one explanation technique in their model [24,32,34,38]. All studies used interviews and/or focus groups, with some also conducting cognitive walkthroughs [23,33], think-aloud protocols [24,28], and questionnaires/ surveys [25,27,28,32,33], involving health professionals from a range of specialties such as anaesthesiology, oncology, critical care, and dietetics.

**Table 1:** Characteristics of included studies.

Author (year)	Country	Setting	Intervention	Stage of development/ implementation	Integrated with EHR	Explanation method	Method(s)	Participants
Abraham^a^ (2023) [23]	USA	Quaternary university hospital	Predicts risk of postoperative complications (case studies focused on AKI and delirium)	Prototype	Yes	SHAP	Cognitive walkthrough and interview	Anaesthesiologists, surgeons, certified registered nurse anaesthetists (CRNAs), registered nurses (RNs), and critical care physicians (n = 17)
Anjara (2023) [24]	Spain	University hospital	Predicting relapse likelihood of patients with lung cancer	Prototype	Yes	SHAP, example based explanations	Think aloud	Oncologists (n = 10)
Barda (2020) [25]	USA	Paediatric intensive care, children’s hospital	Predict in-hospital mortality for paediatric ICU patients	Prototype testing (low fidelity)	No	SHAP	Focus groups, guided review and questionnaire	Paediatric critical care physicians and nurses (n = 21)
Besculides (2023) [26]	USA	Hospital network (6 hospitals)	(MUST)-Plus: Predicts hospital patients at risk of malnutrition	Post-implementation	Yes (Epic)	Feature importance	Semi-structured interviews	registered dietitians (n = 17) with 1–15 years experience across 5 hospitals, all female
Bienefeld (2023) [27]	Switzerland	University hospital (Neurosurgical intensive care)	Predict delayed cerebral ischemia in patients with aneurysmal subarachnoid haemorrhage	Prototype testing (low and high fidelity)	No	SHAP	Focus group, survey and interviews	ICU physicians and nurses (n = 95 for the survey); clinicians (n = 3) and developers (n = 3, n = 6 for the focus group); clinicians and developers (n = 11 for interviews)
Fritz^a^ (2024) [28]	USA	Anesthesiology control tower, quaternary university hospital	Predicts post op complications	Prototype testing (high fidelity)	Yes (Epic in test environment)	SHAP	Interviews, think aloud with scenario based testing and SUS questionnaire	Anaesthesiologists, residents, and certified registered nurse anaesthetists (Phase 2 n = 9, phase 3 n = 10)
Helman (2023) [29]	USA	Emergency department and critical care units, tertiary hospital	Risk score of instability	Prototype testing (low fidelity)	Yes	Feature importance	Focus groups	Registered nurses (n = 11), nurse practitioner (n = 1), physician assistant (n = 1), physicians (n = 9)
Henry (2022) [30]	USA	Acute care non-teaching hospital	Targeted Real-time Early Warning System (TREWS) for sepsis (TREWS uses an ML approach to learn patterns from time-series data to predict, in real-time, whether a patient is at risk of developing sepsis)	Post-implementation (6 months prior)	Yes	Feature importance	Interviews	Physicians (n = 13) and nurses (n = 7)
Jauk (2021) [31]	Austria	Regional public hospital	Predicts delirium risk	Post-implementation	Yes	Feature importance	Focus group	Physicians and nurses (n = 15)
Jin (2020) [32]	China	Hospitals (China and USA)	diagnosis and prognosis decisions	Prototype testing (low and high fidelity)	Yes	Feature importance, similar patients	Case studies and semi-structured interviews, and questionnaire	Cardiologist (n = 1), pulmonologists (n = 5), and physicians (n = 3)
Matthiesen (2021) [33]	Denmark	Remote monitoring centre, University hospital	Predicting ventricular tachycardia and ventricular fibrillation (VT/VF)	Near live	Yes	LIME	Cognitive walkthrough, questionnaires and interview (stage 3)	Cardiologist (n = 7)
Pinto (2023) [34]	Portugal	Epilepsy refractory center, hospital	Prediction for EEG seizures	Prototype testing	No	Feature importance, SHAP, LIME, Individual Conditional Expectation (ICE) Plots, Partial Dependence Plots (PDPs), Regression coefficients, Counterfactual explanations	Interviews	Clinicians (n = 10) and data scientists (n = 10)
Samhammer (2022) [35]	Germany	Nephrology	Predicts the risk of infection and graft loss in the next 90 days	Pre-implementation	No	Feature importance	Semi-structured interviews	Junior and senior physicians (n = 14)
Schwartz (2022) [36]	USA	Health system (2 hospitals)	Predicts in-hospital deterioration	Post-implementation	Yes	Feature importance	Semi-structured interviews	Doctors and nurses (n = 17)
Shulha (2024) [37]	Canada	Hospital	Predicts severity of COVID-19 using chest Xray images (and other data)	Prototype testing (low and fidelity)	No	LIME (saliency heatmap)	Interviews and focus groups with scenarios	Physicians (n = 7)
Zilker (2024) [38]	Netherlands	Hospital	Predicts urgency of ICU admission for patients with sepsis	Prototype testing	No	SHAP, Generalized Additive Models (GAMs)	Structured interviews	Clinicians (surgery, trauma surgery, internal medicine, and anaesthesiology) (n = 4)

a Studies were conducted on same XAI model. EHR: Electronic health record, EEG: electroencephalogram, ICU: Intensive care unit, ML: Machine learning, MUST-Plus: Malnutrition Universal Screening Tool, SHAP: SHapley Additive exPlanations, LIME: Local Interpretable Model-agnostic Explanation.

### Quality assessment

The results of the quality assessment can be found in Supporting Information (Table B in S1 Appendix). The majority of studies were assessed as high quality. Almost all studies except Schwartz et al. [36] did not sufficiently describe the relationship between researcher and participants. Several studies lacked detail on the recruitment of participants [32,34,37,38]. Five studies did not provide sufficient detail on their qualitative data analysis process, and this made it difficult to determine the validity of the findings [31,32,34,37,38].

### Results from synthesis of user perceptions

**Fig 2:**
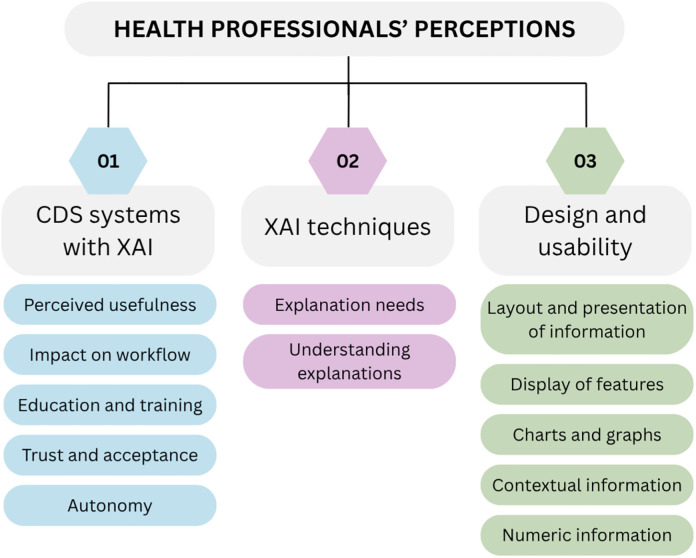
Themes identified from health professionals’ perceptions.

A total of 338 qualitative results were extracted from 16 papers. Data extraction is available in the Supporting Information (Table C and Table D in S1 Appendix). Perceptions of (1) the CDS system with XAI in general, (2) the XAI techniques, and (3) design and usability are presented below and depicted in Fig 2.

#### 1. Perceptions of CDS systems with XAI.

Perceptions on the CDS systems overall were coded into the themes; perceived usefulness, impact on workflow, education and training, trust and acceptance, and autonomy. A summary of these themes, example results and quotations are presented in Table 2.

**Table 2:** Examples of user perceptions from included studies.

Theme	Example
Perceived usefulness	
Identified patients at risk	*“...we’re catching these patients that we wouldn’t necessarily see until...[day] six.” Another RD stated, “I think it has helped us catch a handful of patients who wouldn’t normally maybe been seen high risk.”* [26]
Validate decision or prompted reconsideration	The tool could be useful as an additional data source to confirm an assessment of low risk. [37] *“The AI [artificial intelligence] suggestions are only a bonus that it can either help me…feel confident about my impression or…maybe keep me aware if I’m…missing something.”* [29]
Facilitated teamwork	They additionally described the system as helping to prompt time-dependent actions and to coordinate across multiple care team members. *“I think we try to get them in front of a provider a little bit quicker or get some of the stuff started out in triage.”* [30]
User or context dependent	The admission to ICU is helpful for medical experts with less experience. *“For younger colleagues [...] who don’t have that much experience with [determination of ICU admission] yet, it’s certainly helpful, also just to make sure that nothing is overlooked*” [38]
Increased workload (negative)	*“Sometimes we’re reading through the chart, we’re looking at a patient’s BMI and we don’t think they’re going to be malnourished. So sometimes those patients do have the high score [on the system], but their BMI is very high and we’re reading through the chart and we’re where we have a low suspicion of malnutrition. So sometimes, it can add to the workload in a way that patient is not, probably not very high risk and they could have waited.”* [26]
Saved time	*“Doctors’ time is valuable, [and] quickly estimating the risk of a patient [using the system] is a useful function.”* [32]
Impact on Workflow	
Aligns with workflow	Regardless of their understanding of the ML behind the system, physicians were generally responsive to its alerts and integrated them into their diagnostic process. [30] *“Right now it feels like we have so many screening tools, if there was [the] ability to kind of streamline it to using just one [it would be great].”* [26]
Does not align with context of use	High cognitive effort or attention required to use the model. *“Trying to think about what that actually means…one, you could be wrong, or if it’s 3:00 in the morning…some of the mental gymnastics you’d have to do.”* [25]
Need for further education	*“I wish I knew exactly how it worked or how the algorithm captures these patients.”* [26] *“I would say that I wasn’t given much training as to how I should be using it… I would say that I don’t yet like fully use or think that I can use it to its full potential.”* [36]
Trust and acceptance	
Perceived accuracy	*“For example, the [tool’s] alarm had too many false positives, which gave a lot of extra work and everything, and we actually chose not to use it because there were too many sources of error, and you only really discover that when you work with it [new algorithms].”* [33]
Trust is earned	Not all physicians agreed with the tool’s assessments, but felt that more exposure to a larger number of predictions would be necessary for them to gauge how much they trusted the tool. [37]
Evidence	*“I think, really like a study showing that the score has been used and the evidence behind it...if it’s published and peer reviewed, I think I definitely, personally I’d be more more inclined to use it.”* [36] *“Our documentation isn’t always like right up to date with what’s going on at the moment”.* [36]
Data quality	The score runs at set timepoints during the day, and for some patients, key data may not yet be available, which can result in inaccurate scores. [26]
Alignment with clinical knowledge	The willingness to accept the result of the system also depends on the extent to which it coincides with one’s own thoughts about the specific case. Confirmation, for example, led to a reduction of distrust, whereas differences with the system triggered uncertainty. [35] “*I felt good that it, very much aligned with how the patient was progressing”*. [36]
Autonomy	
Threatens autonomy	*“Of course, it would be a problem if I were to make a decision and the patient knew about the decision of the computer programme or the artificial intelligence and it was different from what I would recommend. I think that would be a problem.”* [35]
Overreliance and deskilling	*“I think [that] there are a lot of people, frankly, that will quickly default to having a tool tell them what to do and stop assessing, and I hope that’s not true, but I’ve seen it happen.”* [30]

*Perceived usefulness.* The theme perceived usefulness was identified in 13 papers, where participants reported the how the systems impacted their work and associated tasks [23,24,26,28–33,35–38].

Across these studies participants commonly reported that the XAI tools support task completion [23,26,28,29,31,33]. For example, XAI systems were seen as helpful in identifying patients at risk of deterioration or complications [23,28,29,31] or in prioritising which patients should be seen first [26,33].

Health professionals also described XAI systems as useful for supporting clinical decision making [29,31,33,35–37]. They were perceived to either validate existing decisions or prompt reconsideration. For example, Mathiesen et al. reported *“the prediction tool was helpful and increased their confidence in their choice of clinical action”* [33].

XAI systems were also perceived as useful for teamwork, [23,30] by coordinating care or improving communication between teams, for example *“…improving the quality of information transmission at handoff”* [23].

Perceived usefulness was reported to be user or context dependent [24,31–33,36,38]. XAI systems were considered particularly beneficial for less experienced doctors [32,38], during night shifts [36], when patient information was incomplete [33], or when patients were unable to communicate [31].

Eight studies linked perceived usefulness to the tool’s impact on workload [26,29–31,33,35,37,38]. In some cases, XAI systems reduced workload. For example, Shulha et al. [37] reported their tool was useful in *“planning for potential ICU admissions over time and helping manage staff resource issues”*. Conversely, in Besculides et al.’s [26] study the XAI tool was already implemented and was perceived to increase workload as it identified more high-risk patients needing review.

Perceptions regarding efficiency or saving time were reported in six studies [23,28–30,32,33]. Participants valued features that streamlined interpretation and decision-making. For example, Fritz et al. [28] reported participants wanted *“display formats that minimize the energy and time spent interpreting data”*.

*Impact on workflow.* Impact in workflow related to how XAI systems aligned with and influenced work processes, and arose as a theme in 9 papers [23–27,30,31,33,35]. Health professionals positively perceived, and were more likely to use, XAI systems that fit into their existing workflow [23–26,30,31,33,35]. For example, participants wanted the XAI tool to be incorporated into existing decision making processes, [35] and require action at the relevant time so they do not create additional work [25]. Similarly aligning the XAI tool with existing systems such as embedding it into the hospital electronic health record was important [23,26,35]. Considering context and users was also reported, with some health professionals having limited time to interpret model explanations and make a decision [23,25,27,32]. For example, the XAI system in Barda et al.’s [25] study was used to predict in-hospital mortality for paediatric ICU patient. Therefore the context required rapid decision-making and participants reported they did not have time for extensive interaction with the system.

*Education and training.* Education and training was a theme that arose in 9 studies, where participants described their need for, or experience of, learning how to use the XAI system [23,25,26,28–30,32,35,36]. In three studies of XAI tools that were already implemented, some participants lacked understanding of the model or features [26,30,36]. In two studies of XAI prototypes, participants perceived if they received further training or practice using the tool it would have increased their understanding [23,32]. Helman et al. [29] reported it took a significant amount of time to explain how to use their XAI tool, which was viewed as a barrier to use in practice. Besculides et al. [26] also found depth of understanding differed for those who were trained by data science team and those who were trained by supervisors or managers.

*Trust and acceptance.* Health professionals’ trust and acceptance of XAI models were reported to be influenced by performance, data quality, evidence, and alignment with clinical knowledge.

Perceptions on the model’s performance was reported in 13 studies [23–26,29–37]. Participants in several studies described the role of the model’s performance in influencing trust [23,25,31–33,36]. For example *“The more accurate it is, in my opinion...the more trust I have in the tool”* [36]. Participants also had concerns with performance regarding unique patients believed to be outliers [25,26,29,36]. A number of studies also reported trust could be earned over time as model performance was proven [23,25,31–33,36], for example, an electrophysiologist said *“I just think I should see that it confirms our decisions in enough cases - then I would feel comfortable about colleagues leaning on it”* [33].

Several studies reported trust and acceptance of model outputs were facilitated by providing evidence for the model effectiveness on patients and/or evidence of the output [23,29,30,33,35,36]. For example, participants in Abraham et. al.’s [23] study reported risk thresholds shown in the bar graph explanation appeared arbitrary and should be based on prior empirical studies. Similarly, perceptions of the XAI model’s data quality impacted participants trust and use of tools and trust in outputs were reduced when XAI system’s used hospital data that participants knew was unreliable [23,25,26,29,32,33,36].

XAI model predictions were reportedly more likely to be accepted when they aligned with health professionals’ knowledge, or with the patient context [23,25,27–30,32–36] This applied to explanations as well, for example, “*If the algorithm keeps showing me a new biomarker for which I have no clue that it has an influence on DCI [delayed cerebral ischemia], it makes me wonder is [the system] just spitting out utter nonsense or did we [clinicians] just not know about this?”* [27]

Health professionals’ involvement in the development also arose as a theme. [30,36]. Participants valued asking questions about design choices and providing input [30], and wanted to “*know that clinicians had been involved in the development of the system or that clinicians would have the opportunity to provide feedback on system performance after its implementation*” [36].

*Autonomy.* Five studies included perceptions related to health professionals’ autonomy [26,29,30,33,35]. Participants wanted to exercise their professional judgement and override the XAI recommendations when perceived to be inaccurate [26], and were concerned *“that regulatory agencies might use these systems to standardize care even in scenarios where a clinician disagrees with the system, potentially leading to overtreatment and patient harm”* [30].

Participants also raised concerns that the XAI system could lead to over-reliance and reduce critical thinking skills [29,30,33,35]. For example, *“more novice practitioners might just see the recommendations on the screen and…automatically follow them without necessarily… applying what the recommendation is to the clinical context and…gauging independently whether or not that’s appropriate”* [26].

#### 2. User perceptions of the XAI techniques.

Perceptions related to the XAI technique were coded as explanation needs, and understanding explanations.

*Explanation needs.* Perceptions related to why the AI system needed to be explainable or what users wanted from explanations were classified as explanation needs.

The level of understanding users desired varied. In two studies participants reported they were not interested in understanding the model itself, but rather the output [27,35]. For example a resident physician reported *“I don’t really need to know how the algorithm works. What I need to know is …which factors and which values do I need to look at and why”* [27]. Conversely, in two studies some participants reported wanting to also understand the model at a global level [25,36], however Schwartz et al. [36] found this need was user dependent, with healthcare professionals that had more knowledge of predictive modelling wanting more information.

Validation of the model output was a major reason for explainability, and was reported in nine studies [25–27,30,32,33,35,36,38]. For example, Besculides et al. [26] reported dieticians checked factors contributing to the score when they questioned the prediction. Both Zilker et al. [38] and Schwartz et al. [36] reported that understanding the prediction was important for trust, and Samhammer et al. [35] found that when the rationale for the recommendation could not be understood, physicians would revert to their own assessment and not rely on the model.

Eight studies in this review reported participants desired actionable information from the XAI systems [23–25,28,30,32,36,37]. In addition to a prediction and explanation, participants reported wanting recommendations for next steps to take based on the output. With regard to feature based explanations, participants in three studies reported wanting to see the variables that could be altered through interventions [23,25,28,32]. For example a participant said *“What you want are modifiable things... History of hypertension? You’re not going to be able to do anything about that”* [21].

Health professionals in three studies reported XAI explanations could help communicate risks or escalations in care to patients [23,35,37]. In contrast, in Besculidies et al’s [26] study of a system to predict patient risk of malnutrition, participants reported that they did not mention the prediction score to patients and communication with patients remained the same post-implementation.

Additionally, explanations were wanted by users for educational purposes, for example, so health professionals could learn from the model [25]. Explanations were also viewed as useful when conducting research [24].

*Understanding explanations.* The explanation methods used are described in Table 1. Of the four studies that implemented multiple explanation techniques within their models [24,32,34,38], only one directly examined participants’ perceptions of the different methods [34]. The study was unable to determine a preferred explanation approach, likely because of the clinical context where there was a limited understanding of the electroencephalogram (EEG) mechanisms that occur prior to a seizure.

Five studies [23,25,27,28,34] specifically described using SHapley Additive exPlanations (SHAP), and four studies reported participants had difficulties understanding SHAP plots or SHAP values. For example in Zilker et al.’s study which presented four interpretation plots to clinicians, participants said the SHAP plots were *“confusing and not really self-explaining”* because there are *“too many dots in the plot*”. Similar results were reported in Bienefeld et al.’s study, with a participant referring to the SHAP values of dynamic contributors saying *“I don’t need to know all this, I am not a mathematician”.*

Local Interpretable Model-Agnostic Explanations (LIME) were also used by models in three studies [33,34,37], however, participants only described its usefulness in one. Shula et al. [37] used LIME to highlight chest x-ray images to show areas of highest contribution to the prediction. Participants valued the option to toggle the explainability overlay, as it helped them assess how consistently the tool identified elements of the x-ray image that could contribute to the overall disease severity. However, it was unclear to participants how the consolidation in the image impacted the severity score and how the severity score influenced the output (i.e., the risk of ICU admission).

Example-based explanations were used in two studies [24,32]. In Anjara et. al.’s [24] study the majority of participants found the examples confusing and felt the system was simply retrieving similar patient records without revealing how the model actually analysed the data. In Jin et al.’s [32] study two participants reported highlighting similar patients and their disease progression was very informative, and helped to validate predictions.

#### 3. Design and usability.

Health professionals’ perceptions involving design and usability were found in 14 papers [23–34,37,38]. From these perceptions design recommendations were developed (see Table 3).

**Table 3:** Design recommendations for XAI systems based on health professionals’ perceptions.

Design recommendation	Study	Example results from studies (participant quotes italicized)
Layout and presentation of information		
Show high-level information with details available on demand	[23–25,28]	Clinicians recommended using pop-ups or tabs to offer additional information, so clinicians could choose how much information to read. [23]
Avoid complex or multi-page displays that slow down decision-making	[23,24,29,32,33]	*“If it’s easily presented and you don’t have to go in and look through 4 pages and such and if it was on the front page and brought up “number of episodes”* [33]
Use colour coding to indicate level of risk or severity -Ensure thresholds are consistent across settings [28]	[23,28,31]	*“We’re all so color sensitive. … So like for me, by including red stuff on there, my brain just knows, I keep thinking I need to look at that first.”* [23] *“I expected the red to be on top, but it’s the higher value that’s on top. So we have—I think that needs a little differentiation for me. So the color provides one piece of information and the numeric provides the other?”* [28]
Use simple terminology, clear labels, and avoid unclear abbreviations	[25,28,33]	The naming of the parameters were sometimes found difficult to interpret [33] *“What is this PPM? Is it permanent pacemaker?”* [28]
Allow users to customize display formats based on their role or workflow	[23,30]	Clinicians also valued that they were able to… customize the interface and alert sensitivity to their environment and patient population. [30]
Avoid narrative text formats for critical data	[23,32]	*“Tables, grids or graphs, in general, are better [than paragraphs].”* [32]
Display of features		
Aggregate information to reduce information processing. E.g. grouping features by similarity, time of measurement	[23,25,28]	*“I really appreciate the groupings on this graph…I said well there’s only an 8% risk of death but it’s being driven largely by this neuro bucket, so what’s going on there?”* [25]
Present features in descending order of added risk	[23,24]	Participants highlighted the lack of order of priority in patient attributes displayed in the explanation. The important attributes contributing to the relapse score was not displayed in an intuitive way. [24]
Omit features making very small contributions	[28]	*“Let’s say there was a less than 1% [contribution]. And then I could click it here and it would show me all the other stuff that’s in the model. I think I’d be able to better focus on these things.”* [28]
Indicate directionality for trend-based features	[25]	*“It would be nice if I could just say, ‘pulse ox is decreasing, creatinine is increasing’”* [25]
Charts and graphs		
Use simple to interpret plots to understand at a glance what features impacted the prediction	[24,25,27,33,34,38]	*“But this information over here [referring to Shapley values of dynamic contributors,* Fig. 2E*], I don’t need to know all this, I am not a mathematician”* [27]
Use plots for visualization of trend-based data	[25,28]	*“For some reason, I look at the graph and I can assimilate that information quicker, and it requires less brain cells to digest.”* [28]
Use tables and/or bar charts for visualization of risk	[23,31]	*“Tables, grids or graphs, in general, are better [than paragraphs].”* [23]
Display explanations in different formats to suit various users	[25]	*“Is it possible that when you click on lactate results it could both bring up the raw data graph as well as the little components of the lactate table?”* [25]
Contextual information		
Provide clinical context information alongside explanations	[23,25,27,32]	To establish that context, clinicians accessed additional patient specific information (patient history, laboratory values, diagnostic tests, imaging, etc.) from existing EHR systems in parallel to using the DCIP. [27]
Ensure there is a clear distinction between contextual information and the explanation	[37]	...physicians erroneously assumed that the additional data present in the tool, namely vitals and laboratory values, were being included in the x-ray severity score. [37]
Numeric information		
Express risk as percentage (reduced information processing)	[23,25]	*“I like seeing the percentage because it would enable me to compare this patient to other patients more easily. And I think that most people would have a sense about whether 13% chance of AKI– especially if they get these [tool] reports for many patients– they’ll have a sense of if that’s a low or a high number.”* [23]
Express percentages as whole numbers - Percentages with decimals created uncertainty - Percentages expressed as negative values and percentages that did not add up to 100 created confusion	[28,33]	*“Yes, I think again that if you present 58.6% then it expresses an accuracy that you may have difficulty navigating with. I know it from other areas in the medical world, the thing about expressing something with a decimal number, it expresses an accuracy for which there may be no evidence at all […] I have a hard time relating to the number […] it’s problematic to translate that into something clinically relevant.”* [33]

Regarding the layout and presentation of information, health professionals reported the need to present concise, high-level information with the option to access additional detail on demand and avoid complex displays. Colour coding was viewed as a helpful visual cue for conveying risk severity.

For presentation of features, healthcare professionals preferred grouping features and ordering them by their contribution to predicted risk to allow easy and quick interpretation.

Simple, interpretable visualizations, such as bar charts and line graphs, were preferred for understanding predictions at a glance. Flexibility in presentation formats was also valued, enabling alignment with individual user preferences.

Health professionals valued the presentation of contextual information such as patient results alongside predictions and explanations, and when this was not included in XAI systems they had to access this information separately when making their assessments.

For numeric information, health professionals reported preferring risk predictions expressed as whole-number percentages, while decimals, negative values, and outputs that did not sum to 100% were viewed as confusing and potentially misleading.

## Discussion

This scoping review revealed that healthcare professionals perceived CDS systems with XAI as useful for supporting work processes, clinical decision making and teamwork. Acceptance and use were dependent on factors such as integration into workflows, performance, data quality, and the alignment of outputs with existing clinical knowledge. Regarding explanation techniques, health professionals wanted XAI systems to provide clear, actionable, and patient-relevant explanations, primarily to validate predictions and guide clinical decision making. Some explanation techniques were difficult to interpret, particularly SHAP values and visualizations. Participants preferred explanations that presented concise, high-level information with simple, interpretable visualizations, clear risk indicators like colour coding, and access to contextual patient data.

Next to perceived benefits of XAI CDS systems, factors such as workflow integration, system performance, education and training, and impact on clinician autonomy emerged as key issues influencing their uptake. Many of these themes are long accepted requirements for CDS as outlined by Bates et al. in 2003 [39], and are not unique to XAI or hospital settings as described in a review of AI adoption in healthcare [40]. However, our review found aspects that were specific to XAI explanations. For example, the inclusion of explanations often required additional training and time to interpret, which can further impact workflow, particularly in acute care. Several studies also identified that participants were concerned about over-reliance and a potential decline in critical thinking skills. These concerns are supported by prior findings that explanations can foster unwarranted trust in AI, prompting healthcare professionals to follow recommendations even when inappropriate [14,41]. Alignment with users own clinical knowledge was key to acceptance. Bienefeld et al. explored this, identifying opposing mental models between developers and clinicians, with developers using XAI to discover new knowledge and clinicians to confirm existing knowledge [27]. Several studies in the current review found participants accepted and trusted explanations when contributing features were consistent with their clinical knowledge.

There are several motivations for incorporating explainability, including to control system behaviour, improve model performance, discover new knowledge, and to meet legal and regulatory requirements [4,5]. Regulatory status was not mentioned in the included papers, so we do not know if this was a factor in these studies. This review found that health professionals often viewed explanations in CDS systems as tools not only for validating predictions but also for identifying actionable insights. For instance, clinicians commonly used the contributing features in explanations to determine which variables could be modified to influence patient outcomes. However, most models and explanations were not designed to be used in this way. This may suggest that there is a need for the product developer to improve the communication of the intended use of the system, in order to ensure that the scope of its use and limitations are clearly understood by users. However, this explanation alone may not be sufficient. Prior research on XAI across industries, has also found that the perceived quality of explanations is closely tied to their actionability and practical relevance for end users [8]. This suggests that an explanation of model limitations without suggested actions might not be perceived as useful. The XAI-evaluation checklist by Brankovic and colleagues [42] recommends assessing, reporting and discussing the actionability of explanations. This reiterates the importance of designing systems with health professionals. Additional interface features may be necessary to guide users toward effective next steps in patient care and increase acceptance and use.

Explanation techniques, and the design and usability of explanations, all influence how health professionals interpret outputs of CDS systems. Across the studies included in this review, feature importance and in particular SHAP were commonly used. SHAP values and plots were found to be challenging to interpret. Several design recommendations were also identified in this scoping review to support practical application. We recommend developers consider the following:

(1) Show high-level information with details available on demand and avoid complex displays
(2) Use colour coding to indicate level of risk or severity and simple, interpretable visualizations such as bar charts and line graphs
(3) Present features in descending order of added risk and group related information to reduce information processing
(4) Provide clinical context information alongside explanations while ensuring a clear distinction from the explanation
(5) Express risk as whole-number percentages to reduce confusion

Prior research has shown that certain cognitive biases can be amplified by specific explanation styles and designs [43] and that providing too much detail may reduce user performance due to information overload [44]. Design may be an even more important consideration in a hospital setting where rapid decision making is often required. Our findings highlight the need for a careful balance between interpretability and usability, and the importance of user-centred design and iterative testing in developing explanations that genuinely increase understanding and support effective clinical decision-making.

This review has several limitations. First, despite the use of a systematic search strategy across multiple databases, three relevant studies were identified only through reference list screening and not via the database search. These omissions highlight the challenges of identifying literature in an emerging and inconsistently defined field. For example, one study did not include any of the terms from our qualitative category in its title, abstract, or keywords [24], while two studies described their systems using general ML terminology without referencing explainability or interpretability [23,30]. As such, the search strategy may have missed other relevant studies that used alternative terminology, potentially limiting the comprehensiveness of the review. Additionally, while the design recommendations are useful for developers, the results are preliminary and higher level of evidence is needed to make strong recommendations. Although a quality appraisal was conducted using the CASP checklist [22], no studies were excluded or weighted based on quality. Furthermore, some included studies did not focus primarily on explainability or user perceptions, instead reporting these findings as secondary outcomes. As a result, the depth and richness of the qualitative data varied, potentially limiting the strength of the conclusions drawn.

## Conclusion

This scoping review showed healthcare professionals perceived CDS systems with XAI as useful for completing tasks, clinical decision making, and supporting teamwork. Systems need to be integrated into workflows, perform optimally with reliable data, and align with existing clinical knowledge in order to be valuable in clinical practice. This review also highlights that explanation techniques must be designed with health professionals in mind, offering concise, relevant, and actionable insights to support informed clinical decisions. Many participants found explanations difficult to interpret, particularly SHAP values and visualizations, highlighting the need for more intuitive and clinically meaningful explanation formats. If poorly designed, XAI explanations may undermine understanding or add to the cognitive burden in high-pressure clinical settings. As XAI continues to evolve, future research should prioritise optimising explanations to ensure health professionals appropriately trust XAI CDS systems and feel confident in their clinical decision making.

## Supporting information

S1 File PRISMA Checklist. (PDF)

S1 Appendix Search terms, CASP quality assessment, and data extraction. (DOCX)
