# Supplementary material for: Explainable AI in hospital clinical decision support systems: A scoping review of healthcare professionals’ perspectives
Source: PLOS Digit Health. 2026 May 14;5(5):e0001417. doi: 10.1371/journal.pdig.0001417 (PMC13175488; doi:10.1371/journal.pdig.0001417)
Supplement: S1 Appendix — (DOCX) [file pdig.0001417.s002.docx]

**Appendix**

**Table of Contents**

[Table A. Search terms 2](#_Toc227916346)

[Table B. CASP quality assessment^1^ 3](#_Toc227916347)

[Table C. Data from extraction and analysis - general 4](#_Toc227916348)

[Table D. Data from extraction and analysis – design and usability 29](#_Toc227916349)

[References 36](#_Toc227916350)

[Abbreviations 37](#_Toc227916351)

## Table A. Search terms

|  | **Keywords (*.ti,ab,kw,kf.)*** | **MeSH** | **Emtree** |
| --- | --- | --- | --- |
| Explainable AI | explainable or explainability or interpretable or interpretability or understandable or understandability or comprehensible or comprehensibility or intelligible or transparent or human centered or sociotechnical |  |  |
|  | AND |  |  |
|  | machine learning or artificial intelligence or AI or prediction model or predictive model or deep learning or neural network | Artificial Intelligence | Artificial intelligence  Machine learning |
| AND | | | |
| Hospital Setting | hospital* or clinician* or prescrib* or doctor* or nurse* or physician* | Hospitals  Inpatients | Hospital  Hospital patient |
| AND | | | |
| Qualitative | view* or opinion* or accept* or facilitator* or barrier* or perspective* or usability or interview* or observation* or scenario* or survey* or questionnaire* | Attitude to Computers  Qualitative Research  Interview  Focus Groups  Surveys and Questionnaires | Attitude to Computers  Qualitative Research  Interview  Surveys and Questionnaires |

## Table B. CASP quality assessment^1^

| CASP checklist items | Abraham^2^ | Anjara^3^ | Barda^4^ | Besculides^5^ | Bienefeld^6^ | Fritz^7^ | Helman^8^ | Henry^9^ | Jauk^10^ | Jin^11^ | Matthiesen^12^ | Pinto^13^ | Samhammer^14^ | Schwartz^15^ | Shula^16^ | Zilker^17^ |
| --- | --- | --- | --- | --- | --- | --- | --- | --- | --- | --- | --- | --- | --- | --- | --- | --- |
| Section A: Are the results valid? |  |  |  |  |  |  |  |  |  |  |  |  |  |  |  |  |
| 1: Was there a clear statement of the aims of the research? | Yes | Yes | Yes | Yes | Yes | Yes | Yes | Yes | Yes | Yes | Yes | Yes | Yes | Yes | Yes | Yes |
| 2: Is a qualitative methodology appropriate? | Yes | Yes | Yes | Yes | Yes | Yes | Yes | Yes | Yes | Yes | Yes | Yes | Yes | Yes | Yes | Yes |
| 3: Was the research design appropriate to address the aims of the research? | Yes | Yes | Yes | Yes | Yes | Yes | Yes | Yes | Yes | Yes | Yes | Yes | Yes | Yes | Yes | Yes |
| 4: Was the recruitment strategy appropriate to the aims of the research? | Yes | Yes | Yes | Yes | Yes | Yes | Yes | Yes | Yes | No | Yes | No | Yes | Yes | No | No |
| 5: Was the data collected in a way that addressed the research issue? | Yes | Yes | Yes | Yes | Yes | Yes | Yes | Yes | Can't tell | Yes | Yes | Yes | Yes | Yes | Yes | Yes |
| 6: Has the relationship between researcher and participants been adequately considered? | Can't tell | No | No | No | No | Can't tell | No | Can't tell | No | No | No | No | No | Yes | No | No |
| Section B: What are the results? |  |  |  |  |  |  |  |  |  |  |  |  |  |  |  |  |
| 7: Have ethical issues been taken into consideration? | Yes | Yes | Yes | Yes | Yes | Yes | Yes | Yes | Yes | No | Yes | Yes | Yes | Yes | Yes | No |
| 8: Was the data analysis sufficiently rigorous? | Yes | Yes | Yes | Yes | Yes | Yes | Yes | Yes | No | No | Yes | Can't tell | Yes | Yes | No | No |
| 9: Is there a clear statement of findings? | Yes | Yes | Yes | Yes | Yes | Yes | Yes | Yes | Can't tell | Can't tell | Yes | No | Yes | Yes | Can't tell | Can't tell |
| Section C: Will the results help locally? |  |  |  |  |  |  |  |  |  |  |  |  |  |  |  |  |
| 10: How valuable is the research? | Yes | Yes | Yes | Yes | Yes | Yes | Yes | Yes | Yes | Yes | Yes | Yes | Yes | Yes | Yes | Yes |
| Appraisal summary | High quality | High quality | High quality | High quality | High quality | High quality | High quality | High quality | Data collection and analysis not described sufficiently | Low participant numbers, recruitment not described, analysis unclear, ethics not described | High quality | Analysis unclear, recruitment not described, qualitative results are not explicitly described and no quotes are presented | High quality | High quality | Recruitment and analysis not described | Recruitment and analysis not described, ethics not described |

## Table C. Data from extraction and analysis - general

| **First author** | **Method** | **Participants** | **Stage of development** | **Theme** | **Result (perceptions)** | **Example quote (if available)** |
| --- | --- | --- | --- | --- | --- | --- |
| Abraham^2^ | Cognitive walkthrough and interview | all | prototype | Knowledge base | Several clinicians felt reassured by their alignment with the model predictions [when their manual risk ranking was compared to the ML model risk ranking] | “The fact that the one I was concerned about was delirium and that is the one that is being marked as above the threshold is a good overall level of agreement on a basic level (Clinician 5 (C5), Anesthesiologist).” |
| Abraham^2^ | Cognitive walkthrough and interview | All | Prototype | Understanding the explanation | Participants found prediction accuracy statistics for risk estimates in the table (Fig. 1a) valuable as interpretive context (n = 6). | “I like the accuracy. Because if something has a really low accuracy, but the predictive likelihood is high, like, I’ll kind of question, you know, how likely in my mind that is, but… Trying to know how accurate this prediction could be… helpful (C2, Anesthesiologist).” |
| Abraham^2^ | Cognitive walkthrough and interview | all | prototype | Performance (model) | some described the numbers as challenging to interpret and intimated that displaying low prediction accuracy could lead to poor model and tool trust (n = 3). |  |
| Abraham^2^ | Cognitive walkthrough and interview | All | Prototype | Understanding the explanation | However, some described the numbers as challenging to interpret (n=3) |  |
| Abraham^2^ | Cognitive walkthrough and interview | all | prototype | Evidence | The bar graph’s (Fig. 1b) risk thresholds garnered mixed feedback: the use of thresholds was helpful (n = 10), but threshold settings appeared arbitrary. Some felt thresholds should be based on prior empirical studies of clinically relevant thresholds. |  |
| Abraham^2^ | Cognitive walkthrough and interview | All | Prototype | Suggestion for improvement | A few participants recommended showing confidence intervals rather than the accuracy statistic (n = 4). | “You could consider showing a confidence interval, maybe using an error bar on the bar chart. The things that are most relevant at this moment for the user are the positive predicted value and the negative predicted value if you’re dichotomizing. And if you’re just interpreting it as continuous, then having the confidence interval… on the prediction would be useful (C5, Anesthesiologist).”. |
| Abraham^2^ | Cognitive walkthrough and interview | all | prototype | Impact on workflow | [RE The bar graph’s risk threshold] Clinicians also noted that choosing these cutoffs required balancing alert fatigue with detection sensitivity. | “If you set them too low, you’ll get way more alerts than might be clinically present. And you’ll likely get fatigued and potentially [ignore alerts]. If you set them too high, you make it unlikely that any of these would ever be prevented, because you’re apt to just… have them develop (C12, anesthesiologist).” |
| Abraham^2^ | Cognitive walkthrough and interview | all | prototype | Knowledge base | there was mixed feedback about how intuitive some factors were as predictors of each complication. Some factors, such as patient age and creatinine levels, were expected contributors to AKI or delirium risk and stood out to clinicians in their assessment. Others, such as ASA, platelet count, and plateau pressure, were not reviewed during manual evaluations – although factors like urea nitrogen and phenylephrine use were ultimately deemed critical to assessing risk. | “you wouldn’t find me looking at alkaline phosphate to see about somebody postoperative risk of AKI… That’s just way out there (C10, Surgeon).”.; “I think one feature that I’m surprised is not here is the duration of the surgery because that’s definitely something that I think about as important for AKI (C5, Anesthesiologist).”. |
| Abraham^2^ | Cognitive walkthrough and interview | all | prototype | Actionability | A subset of clinicians suggested only displaying modifiable risk factors (n = 10), noting that non-modifiable risks like respiratory factors (n = 9), platelet count (n = 7), and plateau pressure (n = 6) added little usable information for planning postoperative risk management. | “In general, [certain non-modifiable risk factors] are reactive to the patient’s… physiology during the procedure. There could be situations where clinicians maybe manage patients differently in controlling some of these things… But that’s… fairly unlikely. So I don’t really think they really help that much in terms of changing management (C1, Anesthesiologist).” Another clinician thought, “There’s only so much one can do to prevent pneumonia, for instance… that isn’t already being done… I wonder… what’s modifiable and what’s not (C4, CRNA).”. |
| Abraham^2^ | Cognitive walkthrough and interview | all | prototype | Impact on workflow/ Suggestion for improvement | Regarding the mode of tool delivery, there were several suggestions to implement the tool within the Epic EHR |  |
| Abraham^2^ | Cognitive walkthrough and interview | all | prototype | Impact on workflow | When asked how to facilitate tool adoption, several clinicians said that buy-in would increase with an easy-to-access (n = 8) and simple-to use (n = 4) tool. | “The more that you have to do like special web addresses or another email or another password, I think you start creating barriers (C9, Anesthesiologist).” |
| Abraham^2^ | Cognitive walkthrough and interview | all | prototype | Impact on workflow | Considering the time constraints on postoperative handoffs, clinicians stressed that the tool needed to be efficient for quick interpretation and reporting. |  |
| Abraham^2^ | Cognitive walkthrough and interview | all | prototype | Education and training | Incorporating training and education on ML model interpretation would also aid clinicians in understanding the risk report and increase the use of the tool. | “If I was trained and familiar with the graph… I think that would probably be quicker and easier to use (C4, CRNA).” |
| Abraham^2^ | Cognitive walkthrough and interview | All | Prototype | Suggestion for improvement | Some clinicians wanted risk estimates to be computed relative to a cohort with similar characteristics (n = 8). | one clinician felt it is essential to see, “population prevalence or what is deemed to be a concerning likelihood of that complication that makes that one stand out… You might have various complications where… the risk of complication, for example, is like 15 %. Oh, wow, that’s a lot, but it may be that’s actually the average. And so, you know, it’d be really good to see like, this is… beyond average, and I think that… tells you where the patients are, prediction is much higher or lower than the average for patients for other patients having the same procedure (C1, Anesthesiologist).” Other clinicians disagreed, asking questions like, “What am I going to do with this data, like how it compares to an average patient if all I care about [is] my patient (C13, CRNA)?”. |
| Abraham^2^ | Cognitive walkthrough and interview | all | prototype | Performance (model) | n=2/13 described strong skepticism that the ML models would produce valid risk estimates. | “the AKI rate was lower than I would have thought it would [be], and I’m not sure if that’s just in terms of the definition of how it’s defined. I still think it’s extremely likely the patient’s going to have a rise in creatinine post-op, so it’s hard for me to too much question my belief in that regard (C1, Anesthesiologist).”. |
| Abraham^2^ | Cognitive walkthrough and interview | all | prototype | Performance (model) | On the other hand, three clinicians expressed trust in the value of the predictions despite their assessments’ disagreement with the model’s estimates. | One expressed fascination with the idea that “things that we as humans don’t put together in terms of risk factors or what’s contributing to it, maybe there is… risk there, and we just don’t know it (C2, Anesthesiologist/ Critical Care Physician).”. |
| Abraham^2^ | Cognitive walkthrough and interview | all | prototype | Evidence | To promote trust clinicians suggested screening predictors for evidence of a clinical association with a complication (n = 4) |  |
| Abraham^2^ | Cognitive walkthrough and interview | all | prototype | Data quality | To promote trust clinicians suggested and making model inputs transparently available for users (n = 5). | “I want to know what pieces of information were included in it… The more of those things that I can see that I know are important, that are not present, the more—the less confidence I would have. And if I do see all the factors which I know are known to be important, then my confidence goes up (C1, Anesthesiologist).” |
| Abraham^2^ | Cognitive walkthrough and interview | all | prototype | Performance (model)/ Education and training | At least one clinician noted that trust would also evolve as users (1) learn how to relate the ML outputs to subsequent clinical decisions and (2) interact with the predictions and see how well they correspond to patient outcomes. | “I want to know what pieces of information were included in it… The more of those things that I can see that I know are important, that are not present, the more—the less confidence I would have. And if I do see all the factors which I know are known to be important, then my confidence goes up (C1, Anesthesiologist).” |
| Abraham^2^ | Cognitive walkthrough and interview | all | prototype | Actionability | many clinicians stressed the importance of actionable risk reporting (n = 10), the ML-augmented tool will require modifiable risk factors and relevant risk mitigation strategies and recommendations | “[The current prototype is] still kind of missing… the action item, right? What should the blood pressure be to decrease risk, right? Or if this patient is already mechanically ventilated, what should the CO_2_ be to decrease risk? (C7, Anesthesiologist).” |
| Abraham^2^ | Cognitive walkthrough and interview | all | prototype | Actionability | Clinicians also felt the tool would be more valuable if it identified risks and provided risk mitigation strategies. | “Generally, I just want to [see that a complication is at] 46 % [risk] and [understand] that’s really high and then click here for mitigation strategies based on evidence that we know about or whatever.”. |
| Abraham^2^ | Cognitive walkthrough and interview | all | prototype | Design | Several clinicians felt torn between display formats, suggesting allowing users to choose between the two most popular (n = 6). | “Everybody perceives things and interprets things differently. So, I could see why people would appreciate different formats… If this was going to be something that’s always in your sidebar on Epic [EHR], you could pick [different formats for yourself] (C11, CRNA).”. |
| Abraham^2^ | Cognitive walkthrough and interview | all | prototype | Design | Although clinicians provided feedback on the presented risk factors across secondary displays, they also pointed out that the case context mattered during interpretation. | a few clinicians recommended that the surgical procedure(s) performed in the current visit (n = 2), comorbidities (n = 5), and current medications (n = 6) should be included alongside each secondary risk factor report. |
| Abraham^2^ | Cognitive walkthrough and interview | all | prototype | Design | Clinicians recommended using pop-ups or tabs to offer additional information, so clinicians could choose how much information to read. | One clinician suggested making “pulmonary embolism… a hyperlink [to] click on and read about what the incidence is out in the wild versus this patient… (C9, Anesthesiologist).” Another clinician agreed, adding, “A hover-todiscover would be really helpful [be]cause you don’t know what the clinician knows, especially early on. And text, even though you don’t want to read—you would never want to just look and be like, then read the text first. It would just be more graph first, and then if I need to supplement my knowledge, then I would like a hover-to-discover (C2, Anesthesiologist/ Emergency Room Physician).”. |
| Abraham^2^ | Cognitive walkthrough and interview | all | prototype | Perceived usefulness (easier to complete task)/ Impact on workflow | Several clinicians strongly advocated using ML at several timepoints along the perioperative spectrum, including preoperative consultation, intraoperative risk management, postoperative OR-ICU handoff reporting, and postoperative care planning. |  |
| Abraham^2^ | Cognitive walkthrough and interview | all | prototype | Perceived usefulness (easier to complete task)/ Patient/ carer impact | Some mentioned that the risk summary could help them communicate risks to patients and other clinician consultants before surgery. | The journey with the patient starts way before the surgery itself - you actually see the patient preoperatively, so you know what the patient actually walks into the clinic with. So, all the preoperative contributing factors is something which we will look out for. And we’ll discuss with the patient. So, to us that that is something which is very, very important (C10, Surgeon).” |
| Abraham^2^ | Cognitive walkthrough and interview | all | prototype | Perceived usefulness (easier to complete task) | Intraoperatively, the tool could prompt clinicians to re-examine patient charts or double-check their own care plans, instilling confidence in their management. | One clinician felt strongly that seeing agreement between their chart review and the predictions would increase their confidence in acting to prevent complications because “it would be a basis for my decision making or a basis for my rationale. Anesthesia is not a definitive practice, and you’ve got to come up with reasons why you’re making decisions. And this would be very helpful when you’re making those decisions (C8, CRNA).”. |
| Abraham^2^ | Cognitive walkthrough and interview | all | prototype | Perceived usefulness (easier to complete task/ decreases time) | Participants also felt the displays would speed up the preparation for OR-ICU handoffs, improving the quality of information transmission at handoff (n = 5) and reminding caretakers of priorities intraoperatively (n = 2) and during postoperative ICU care (n = 2). |  |
| Abraham^2^ | Cognitive walkthrough and interview | all | prototype | Perceived usefulness (easier to complete task) | Using ML in handoffs could also remind OR teams to report on specific complication risks. Several clinicians complained that current handoff templates were not strictly followed, and postoperative reports failed to address patient risks and immediate concerns. | Clinicians expressed concern that “[The ICU team is] not getting a full report. [The OR team is] just giving you know, they have so many patients they have to reporton, they’re not going to include the post-op complications in their report, I don’t think (C13, CRNA).” |
| Abraham^2^ | Cognitive walkthrough and interview | all | prototype | Design | A modest number of clinicians also believed that secondary displays were not necessary for every patient (n = 7) and would not be used by most clinicians since, “on a science level or a research-level, it’s interesting, but clinically, this is not going to give you any new information or be useful at all (C6, Surgeon).” | “I just don’t know how many people are going to have time and desire [to read this secondary display], and how useful is that going to be for clinicians when they’re trying to [take] care of the patient (C13, CRNA).” |
| Abraham^2^ | Cognitive walkthrough and interview | all | prototype | Perceived usefulness (easier to complete task) | In post-handoff care, ICU clinicians anticipated using the displays to validate the reasoning behind their care plan (n = 5) and better recognize at-risk patients without an apparent risk factor (n = 4), citing the ability of computational models to find complex patterns they cannot. |  |
| Abraham^2^ | Cognitive walkthrough and interview | all | prototype | Perceived usefulness | Using algorithms to identify risk factors would educate clinicians on unnoticed or unexpected components of risk mitigation | “it could educate me as a clinician for something that I was unaware of (C14, RN).” Another clinician suggested an example of how a nurse might use the ML-augmented tool to identify AKI. “If you got a nurse in the middle of the night that’s concerned about something, she could go back in the chart… and go, ‘Oh, yeah, the guy is at risk for this’ […] Maybe she doesn’t know what to look for to identify acute kidney injury… She already knows the guy’s at a high risk for an acute kidney injury. […] she would be able to look for certain things to alert a physician if necessary (C8, CRNA).” With algorithm-generated predictions and alerts, the tool could “aid |
| Abraham^2^ | Cognitive walkthrough and interview | all | prototype | Perceived usefulness (easier to complete task) | Respondents felt that RNs (n = 4) and trainees (n = 4) could use the tool best during postoperative care, speculating that it might help improve interdisciplinary shared understanding. A few also noted that the ICU team could use the report during shift handoffs post-transfer or rounds. | As a nurse, maybe they’re not aware of… the rationale for what the doctors are doing or what they should look for (C8, CRNA),” |
| Anjara^3^ | Think aloud | Oncologists | Prototype | Understanding the explanation | Participants (7/10) stated when presented with the relapse score and explanation, they were confused by the influential examples (other patients) that served as the explanation. | “I would like someone to explain it to me more. I know this is very difficult so I won’t understand all the AI explanations. . .[but] I would want someone to explain the method more.” |
| Anjara^3^ | Think aloud | Oncologists | Prototype | Understanding the explanation | In showing the ‘influential examples’, some participants assumed that the system regurgitated information from the electronic medical record system, without adding additional layers of analysis: in other words, the machine learning effort was obscured. | “The table gives information about the patient that I already know. This is information I give to this system. But I want to know why, which are the high-risks [variables].” |
| Anjara^3^ | Think aloud | Oncologists | Prototype | Understanding the explanation | To some participants, example-based explanations did not adequately explain the prediction score. They questioned the prediction score for the complex case as the ‘influential example’ seems to have very different clinical features. | “This is not correct for me. We can’t compare these patients because the type of treatment and type of surgery is different. The prediction [should be] very different for both patients.” |
| Anjara^3^ | Think aloud | Oncologists | Prototype | Perceived usefulness/ Performance (model) | All participants generally acknowledged the potential benefit of the system to assist in decision making, but were skeptical of the current system’s ability to deliver. |  |
| Anjara^3^ | Think aloud | Oncologists | Prototype | Design/ Perceived usefulness | Most participants indicated they were confused by what they were seeing, and half (5 out of 10) indicated they were overwhelmed by the system | “As a clinician, what [is] important to me is the risk of relapse, that’s what I’m going to use. . .the rest provides extra information that we are not all going to use.” |
| Anjara^3^ | Think aloud | Oncologists | Prototype | Design | The mock display indicated that the prediction score was based on a sample of over 1,000 lung cancer patients, it was not intuitive to our participants that the relapse score was based on the entire database of patients, as only two or three ‘influential examples’ were displayed. | “I think the relapse score is important information, but was this patient compared with the 2 other patients here or a bigger group of patients? I think it’s very difficult to do a prediction for relapse only by comparing with a small sample. The idea is very good but it is necessary for us to know the original information from the total group of patients.” |
| Anjara^3^ | Think aloud | Oncologists | Prototype | Design | The added visual of example-based explanations helped participants better understand that the prediction score is supported by data about the patient and other patients | “Here everything is clearer, there’s more data about the patients. It expresses pretty well the differences between them and what they have in common. It seems pretty clear, it’s quite visual. It seems simple.” |
| Anjara^3^ | Think aloud | Oncologists | Prototype | Actionability | Participants found that the system overall “is too general to take out a conclusion” and does not help in deciding “what treatment I would provide.” The example-based explanations were insufficient to feed into a decision-making process. | “If we had a platform with a lot of information, we would use it no doubt. I think it is a good idea and is really necessary.” |
| Anjara^3^ | Think aloud | Oncologists | Prototype | Explanation needs | Some (4 out of 10) of the participants believed that the system was missing something and was not complete in the current form. | “[The system] is missing a lot of things. I need to know why. What is the most important attribute to [indicate] relapse?” |
| Anjara^3^ | Think aloud | Oncologists | Prototype | Perceived usefulness | Some participants thought the current system would be more helpful for research instead of for use in daily clinical work. | “This is more useful for research or for comparing patients but in our daily work it doesn’t provide much information. It provides information to compare patients, it’s more general, not for individual patients. To compare one, two or three patients it’s not very relevant clinically speaking. The example is quite clear but it’s not very relevant in our daily work, to tell you the truth.”– |
| Anjara^3^ | Think aloud | Oncologists | Prototype | Impact on workflow | Many (6 out of 10) participants stated they could not see the system fitting in to their working day. |  |
| Anjara^3^ | Think aloud | Oncologists | Prototype | Actionability/ Suggestion for improvement | Several participants suggested that a dynamic prediction score, with information on how the relapse prediction could be reduced by therapy, would be more useful | “I can see me using this but with more information. I need to know if you put a kind of chemotherapy, something will happen. If you use this type of therapy, you will have better outcomes. [We] need to know something more.” |
| Anjara^3^ | Think aloud | Oncologists | Prototype | Suggestion for improvement | Some participants further noted that a treatment-oriented system, with projected relapse scores based on various therapeutic options, would be preferable. |  |
| Barda^4^ | guided review | some | low fidelity prototype | Actionability | Desire for actionable information, including ways to mitigate risk |  |
| Barda^4^ | Focus groups/ questionnaire | Physicians and Nurses | low fidelity prototype | Explanation needs | Use explanations to verify model information and assess model credibility |  |
| Barda^4^ | Focus groups/ questionnaire | Physicians and Nurses | low fidelity prototype | Explanation needs | Explanation needs influenced by the level of predictive modelling knowledge of a provider. Providers with more detailed knowledge of predictive modelling wanted information about model development processes and how the model compared to similar existing models | Explanation needs influenced by the level of predictive modelling knowledge of a provider. Providers with more detailed knowledge of predictive modelling wanted information about model development processes and how the model compared to similar existing models |
| Barda^4^ | Focus groups/ questionnaire | Physicians and Nurses | low fidelity prototype | Performance (model) | High predictive performance of the model |  |
| Barda^4^ | Focus groups/ questionnaire | Physicians and Nurses | low fidelity prototype | Knowledge base | Predictions aligned with clinical knowledge | “I mean arterial pressure of 250 seems physiologically impossible.” “The leading variables are patient is having respiratory issues and has kidney injury…from a face validity standpoint—yes, that sounds like a patient with a higher risk of dying.” |
| Barda^4^ | Focus groups/ questionnaire | Physicians and Nurses | low fidelity prototype | Performance (model) | limitations in the modelling process (e.g., not accounting for feature correlations) |  |
| Barda^4^ | Focus groups/ questionnaire | Physicians and Nurses | low fidelity prototype | Performance (model) / data quality | Predictions based on outliers, erroneous data, or counterintuitive risk factors viewed negatively |  |
| Barda^4^ | Focus groups/ questionnaire | Physicians and Nurses | low fidelity prototype | Explanation needs | Use explanations to extract knowledge or learn from the model | “Does this model offer new information that I didn’t already have? Like ‘this patient was at high risk for mortality and I didn’t otherwise recognize that.’” “Just telling you what you should know, and what you would appreciate if you clicked into the chart and dove into the information, but at least this is synthesizing that for you.” |
| Barda^4^ | Focus groups/ questionnaire | Physicians and Nurses | low fidelity prototype | Explanation needs | Explanation needs influenced by clinical position and work environment |  |
| Barda^4^ | Focus groups/ questionnaire | Physicians | low fidelity prototype | Explanation needs | Obtain insights to prioritize patients, assess patient statuses, and identify high-risk patients and information of concern | “Can I do anything to mitigate that risk of mortality based on what I know?” “I don’t think there’s a lot I can do about most of that stuff…” |
| Barda^4^ | Focus groups/ questionnaire | Nurses | low fidelity prototype | Actionability | Actionable information, alerts to important changes in patient status, and information to assist in intervention or justify a request for a physician consult |  |
| Barda^4^ | Focus groups/ questionnaire | Physicians | low fidelity prototype | Education and training | Training on use/ interpretation of the model | “One risk, I think, with this type of data presentation, is I are going to over-interpret the results…this is just showing you how the model worked, it doesn’t necessarily mean the model is saying you should act on these specific [factors]” |
| Barda^4^ | Focus groups/ questionnaire | Physicians and Nurses | low fidelity prototype | Design | Clinically irrelevant information (e.g., e.g., a high-risk prediction driven by a low Glasgow Coma Score measurement for a sedated and paralyzed patient) |  |
| Barda^4^ | Focus groups/ questionnaire | Nurses | low fidelity prototype | Impact on workflow | Inappropriate alerts (i.e., relevant and at proper time) adding additional work | “They trigger a sepsis screen every time I do vitals or every 2 hours and you call the doctors and they have to come down and see them and they’re getting really irritated. They don’t want to have to do that, they don’t have time.” |
| Barda^4^ | Focus groups/ questionnaire | Nurses | low fidelity prototype | Impact on workflow | High cognitive effort or attention required to use the model | “If you load it with a lot of numbers that will probably be not helpful…Because it dilutes your attention.” “Trying to think about what that actually means…one, you could be wrong, or if it’s 3:00 in the morning…some of the mental gymnastics you’d have to do.” |
| Barda^4^ | Guided group discussion | doctors and nurses | Low fidelity prototype | Impact on workflow | Large time investment to use the model | “I don’t know if—working on the floor—I get an alert that I have time to go and look through all of this data to try and figure out where the risk is coming from.” |
| Barda^4^ | Focus groups/ questionnaire | Physicians and Nurses | low fidelity prototype | Suggestion for improvement | Incorporating domain knowledge into model, such as including additional relevant predictors (e.g., medications) and defining normal ranges for variables (e.g., setting age- or patient-specific baselines) | “One of the critical things that you might consider finding a way to incorporate into the score is medications.” “I do think, from a model validity standpoint, changing this to include maybe abnormal blood pressure for age, does add a lot.” |
| Barda^4^ | Focus groups/ questionnaire | Physicians and Nurses | low fidelity prototype | Suggestion for improvement | Improving model utility by examining alternative prediction targets, such as morbidity, disease-specific mortality, and event-specific mortality (e.g., mortality as a result of a cardiac event). | “It’d be nice to look at morbidity as well and other things.” |
| Barda^4^ | Focus groups/ questionnaire | Physicians and Nurses | low fidelity prototype | Explanation needs | Some desire for explanations of model processes and at the global-level | “What’s the weight of the data that is available from the moment they did transfer them to the ICU and how does that carry into this predictive model?” “Is it possible to see what the machine learned about the relationship between age and raw numbers of vital signs?” |
| Besculides^5^ | Interviews | Dieticians | Post implementation | Explanation needs | Features contributing to prediction used to understand unexpected or extreme scores | Many RDs stated that they use the hover tool to check the factors contributing to the score when they question it, and often this double-checking enhanced understanding of an unexpected extreme score. |
| Besculides^5^ | Interviews | Dietiticians | Post implementation | Perceived usefulness (easier to complete task) | Predictive score useful in prioritising patients to be seen | “...we’re catching these patients that we wouldn’t necessarily see until...[day] six.” Another RD stated, “I think it has helped us catch a handful of patients who wouldn't normally maybe been seen high risk.” |
| Besculides^5^ | Interviews | Dietiticians | Post implementation | Performance (model) | Prediction score viewed as objective assessment | “The reason the malnutrition predictive model is helpful is because it's pulling objective data basing whether or not they're high risk based on objective criteria that's in the chart.” |
| Besculides^5^ | Interviews | Dietiticians | Post implementation | Performance (model) | Tools accuracy improved over time | “when it first got rolled out, it was not as specific... we were getting a high score on a lot of our patients, but now it's more specific.” Another RD stated, “In general, the model is stronger than when it first started... initially a good portion of the high [predictive] scores end up being malnourished, as opposed to when it started.” |
| Besculides^5^ | Interviews | Dietiticians | Post implementation | Impact on workflow | Improved accucacy resulted in fewer false alarms |  |
| Besculides^5^ | Interviews | Dietiticians | Post implementation | Performance (model)/ Data quality | Human error in data entry was 1 factor that led to an inaccurate score. | Human error in data entry was 1 factor that led to an inaccurate score. |
| Besculides^5^ | Interviews | Dietiticians | Post implementation | Performance (model)/ Data quality | The score runs at set timepoints during the day, and for some patients, key data may not yet be available, which can result in inaccurate scores. |  |
| Besculides^5^ | Interviews | Dietiticians | Post implementation | Performance (model) | Even when data were present and correct, other factors reportedly caused the predictive score to be wrong. | Inaccuracy was noted among patients at weight extremes—either very low (often young adults, elderly, or Asian patients) or very high, as well as among amputees (because BMI calculations are not meaningful in this population). Albumin levels, fluid overload, anemia from a bleed, and low platelets due to a transplant were other factors reported by RDs across hospitals to be associated with false positives. |
| Besculides^5^ | Interviews | Dietiticians | Post implementation | Autonomy | the need for more autonomy to exercise their professional judgment and override situations where the tool may be inaccurate instead of seeing all patients identified as high risk by MUST-Plus |  |
| Besculides^5^ | Interviews | Dietiticians | Post implementation | Education and training | Not everyone understood what the features meant | All RDs had knowledge of the interface’s hover tool (although not all used it) and understood that the factors listed in it were those that contributed the most to the score. ; “I wish I knew exactly how it worked or how the algorithm captures these patients.” |
| Besculides^5^ | Interviews | Dietiticians | Post implementation | Education and training | could articulate a basic understanding of the factors that may contribute to the predictive score, but some, particularly those at Mount Sinai Hospital and clinical nutrition coordinators, seemed to have a deeper understanding of how the tool worked |  |
| Besculides^5^ | Interviews | Dietiticians | Post implementation | Patient/ carer impact | communication with patients did not change with the implementation of the predictive tool and they do not mention the score when they meet with patients | RDs reported that communication with patients did not change with the implementation of the predictive tool and they do not mention the score when they meet with patients.; “[The score] is less defining how long we're spending with the patients and more defining who we are seeing.” |
| Besculides^5^ | Interviews | Dietiticians | Post implementation | Patient/ carer impact | the amount of time they spend with patients was about the same since implementation. |  |
| Besculides^5^ | Interviews | Dietiticians | Post implementation | Perceived usefulness (increase in workload) | Increase in number of patients to see (some cases where workload increased because MUST-Plus misidentified patients as high-risk, even though upon chart review, RDs could tell these patients were likely low-risk. Even though these cases led to additional work, RDs repeatedly noted that they accepted these shortcomings and valued the score overall). | sometimes we’re reading through the chart, we’re looking at a patient’s BMI and we don’t think they’re going to be malnourished. So sometimes those patients do have the high score, but their BMI is very high and we’re reading through the chart and we’re where we have a low suspicion of malnutrition. So sometimes, it can add to the workload in a way that patient is not, probably not very high risk and they could have waited. |
| Besculides^5^ | Interviews | Dietiticians | Post implementation | Education and training | There was a difference in depth of tool understanding between the supervisors or managers who were trained by the data science team and those who supervisors or managers trained. |  |
| Besculides^5^ | Interviews | Dietiticians | Post implementation | Suggestion for improvement | Improving accuracy by changing the factors such as low albumin that feed the algorithm or modifying the weight certain factors play. |  |
| Besculides^5^ | Interviews | Dietiticians | Post implementation | Education and training | more education about how the tool works. | What exactly determines a high score? Do factors like albumin, platelets, and hemoglobin all carry the same weight in the model? |
| Besculides^5^ | Interviews | Dietiticians | Post implementation | Suggestion for improvement | Running the score only when all laboratory data were present was also suggested as an improvement. |  |
| Besculides^5^ | Interviews | Dietiticians | Post implementation | Impact on workflow | a single screening tool would be preferred to the use of multiple tools. Despite an overall belief that the score is valuable, it was noted repeatedly that the score was not used in isolation to diagnose malnutrition. | “Right now it feels like we have so many screening tools, if there was [the] ability to kind of streamline it to using just one [it would be great].” |
| Bienefeld^6^ | Interviews | Clinicians | Prototype (high fidelity) testing | Impact on workflow | the high-risk context and need for rapid decision-making did not allow for extensive system interactivity or the use of advanced analytics tools. | “When [the DCIP] gives me an elevated risk score, I must be able to see within minutes if [the results] make sense and whether I should order an emergency CT, administer an electrocyte transfusion, etc. We don’t have much time to waste by checking for more details. These [patients, they can change [get worse] really quickly”. |
| Bienefeld^6^ | Focus groups | Clinicians | Prototype (high fidelity) testing | Impact on workflow | Context is high risk and rapid decision making is needed: Explanations needs to be quick to interpret | “When [the DCIP] gives me an elevated risk score, I must be able to see within minutes if [the results] make sense and whether I should order an emergency CT, administer an electrocyte transfusion, etc. We don’t have much time to waste by checking for more details. These patients, they can change [get worse] really quickly”. |
| Bienefeld^6^ | Interviews | Clinicians | Prototype (high fidelity) testing | Explanation needs | The model itself must be interpretable for clinicians, but the clinicians found this information unhelpful in increasing their understanding and trust in the system. | “Here [referring to Fig. 2B 1], is the overall risk score of 0.8 and here [referring to Fig. 2C 2], I see what contributes to the baseline risk. That makes sense. But this information over here [referring to Shapley values of dynamic contributors, Fig. 2E], I don’t need to know all this, I am not a mathematician. What I need to know is do these results [referring to Fig. 2B 1] make sense clinically […]. For instance, when I see that on November 30th [Fig. 2E 3], the risk for DCI was high, then I want to know what we did that day; like did we do a CT scan, and did [the patient] actually develop a DCI? Also, if I administered Mannitol [medication to lower intracranial pressure17], would the risk go down then? These kinds of things I need to know to trust [the DCIP].” |
| Bienefeld^6^ | Interviews | Clinicians | Prototype (high fidelity) testing | Knowledge base | Not interested in understanding model. Wanted to understand the clinical plausibility of the included parameters and output data within the clinical context. | “I don’t really need to know how the algorithm works. What I need to know is when [the DCIP] tells me the risk [of an upcoming DCI] is rising, why it is rising. That is which factors and which values do I need to look at and why”. (Resident physician, 98) “All I need to understand is why the risk for a particular patient to develop a DCI has changed over time. To be able to see what these parameters are and if they make sense clinically [referring to Figure 3, E 5].” (Attending physician, 96) |
| Bienefeld^6^ | interview | clinicians | high fidelity UI prototype | Knowledge base | The patient tells the truth. | “[The DCIP] cannot see, hear, or touch the patient. For example, if [the patient] can’t move his arm anymore, this can be really important. [The DCIP] can’t see any of this [clinically relevant information gained from physical examinations]." |
| Bienefeld^6^ | Interviews | Clinicians | Prototype (high fidelity) testing | Design | Provide patient specific sources of information; Clinicians accessed additional patient information from EHR in parallel to using tool | “It would be great if we could enter [information from the physical examination] here [referring to Fig. 2F 4] so we could see how [the neurological deficits] combine with all the other data in the system.” ; To establish that context, clinicians accessed additional patient specific information (patient history, laboratory values, diagnostic tests, imaging, etc.) from existing EHR systems in parallel to using the DCIP. To facilitate such holistic patient assessments, future designs should aim for a complete integration of systems (which was not possible in our case due to a walled-garden design of the existing EHR system18). |
| Bienefeld^6^ | Interviews | Clinicians | Prototype (high fidelity) testing | Knowledge base | clinicians relied on established knowledge from evidence-based medicine to compare against and build trust in the model predictions. | “If the algorithm keeps showing me a new biomarker for which I have no clue that it has an influence on DCI [referring to Figure 3, E 5], it makes me wonder ‘is [the system] just spitting out utter nonsense or did we [clinicians] just not know about this?’. If so, studies should look at this biomarker [e.g., Creatinine] and test if it really has something to do with DCI. Then one might declare the algorithm detected a new biomarker. But until then, I cannot trust it because there is no evidence from clinical studies.” (Attending physician, 109). “If the algorithm tells me, the probability [for a particular patient to develop a DCI] is 70% [referring to the overall risk score of the DCIP combining static and dynamic contributors, Figure 3, B 1] and I look at the patient and the neuromonitoring etc. and I don’t see anything abnormal, I don’t think I would trust [the system]. I might be alert and look more closely at all the information and make my own clinical assessment of the patient again but to take it at face value, no, it would take a long time to have this kind of trust in the system.” (Attending physician, 105); “If a certain biomarker pops up again and again, like here [referring to Fig. 2E 5] but there is no evidence in the literature on this biomarker [i.e., Creatinine] concerning DCI, it is hard to trust [the DCIP]. But what if the machine was indeed correct? Then we would discover a new biomarker and the potential for learning would be huge”. |
| Fritz^7^ | focus groups | physician and nurses | low fidelity prototype | Understanding the explanation | When patients were at high risk for complications, clinicians wanted to know what variables were contributing to the risk | “you have some idea how to address them” |
| Fritz^7^ | focus groups | physician and nurses | low fidelity prototype | Actionability | More interested in modifiable risk factors than nonmodifiable risk factors, because these were “factors that we can control” rather than “factors that we can just be sad about” | One clinician even asked for “a score that’s based entirely on modifiable risk factors to show them how much we could affect things”  “What you want are modifiable things ... History of hypertension? You’re not going to be able to do anything about that.” “Something that might be cool…if you can kind of qualify some factors as modifiable versus nonmodifiable. These patients have the highest degree of modifiability of their risk of AKI…It’s not just because they have a history of CKD. It’s because they’re getting... their MAPs going to be about 57.” |
| Fritz^7^ | interviews | physician and nurses | low fidelity prototype | Actionabiliy | highlight input variables that could be altered through interventions | “it’d be helpful to know if any of them are modifiable” |
| Fritz^7^ | interviews | physician and nurses | high fidelity prototype | Actionability | Would like to see what features cause predicted risk to change over time | “If there is something that one can intervene on, that you are intervening on…and if we’re getting the feedback that we think we should be getting. Maybe that can be captured only with the individual patient views.” |
| Fritz^7^ | focus groups | clinicians | high fidelity prototype | Perceived usefulness (easier to complete task) | Clinicians wanted to identify patients where they can take action to prevent complications | “What you want are modifiable things ... History of hypertension? You’re not going to be able to do anything about that.” Participant 4 (attending), focus group 3 “Something that might be cool…if you can kind of qualify some factors as modifiable versus nonmodifiable. These patients have the highest degree of modifiability of their risk of AKI…It’s not just because they have a history of CKD. It’s because they’re getting... their MAPs going to be about 57.” Participant 16 (resident), focus group 5 |
| Fritz^7^ | focus groups | clinicians | high fidelity prototype | Perceived usefulness (decreases time) | Clinicians want display formats that minimize the energy and time spent interpreting data | “If you get too much information you get information overload and you can’t use it. So, I agree with that, the right amount of information at the right time, whatever that means.” “So having the most relevant information on screen, and then either hovering or clicking on a piece of information to get things like confidence bounds, more details that are not useful immediately but are more useful for understanding.” |
| Fritz^7^ | interviews | physicians and nurses | low fidelity prototype | Perceived usefulness (easier to complete task) | ML display was beneficial for aiding risk estimation | “That is something I struggle with in the ACT tower every time—just applying a risk value to a patient when there’s a number of variables. And I feel like the display the machine learning provided was informative” “It was very nice to see quickly a comparison versus an average patient.” “I feel like I have that kind of gestalt if someone’s going to be okay, but it’s nice to see the numbers. It’s really nice to see the numbers.” |
| Fritz^7^ | interviews | physicians and nurses | low fidelity prototype | Actionability | Clinicians wanted the ML display to tell them more explicitly what actions to take in response to the information . And highlight input variables that could be altered through interventions | “it would be useful for me here for the machine learning to suggest these outcomes rather than me picking them. … It’d be wonderful if the machine suggested things and then we could modify it.”  a desire for the ML to select which complication risks were most important to communicate to the bedside anesthesiology team |
| Fritz^7^ | interviews | physicians and nurses | high fidelity prototype | Suggestion for improvement | would like the model to predict additional targets | “And so these are the only two—from historical data, these would be the only things statistically relevant that we would be predicting?”  “I don’t know, mainly—I mean, I’m a cardiac person, so maybe heart failure stuff. But yeah. Kidney, I think, definitely would be one of my top ones.” |
| Fritz^7^ | interviews | physicians and nurses | high fidelity prototype | Knowledge base | user disagreement with prediction raises questions about accuracy | “So it looks like the risk of postoperative death is highest for a patient undergoing a thyroidectomy over a couple of much bigger procedures. How bizarre.” |
| Fritz^7^ | interviews | physicians and nurses | high fidelity prototype | Education and training | more detail about how predictions are made | “So I just want to know, how do you write this patient’s code? Do you have any algorithm which would give these numbers for the patient, the risk of postoperative death?” |
| Fritz^7^ | interviews | physicians and nurses | high fidelity prototype | Education and training | would like to see disclosure of model limitations | “I think it might be interesting also if you think your model has any limitations or blind spots. It might be good to disclose that here.” - |
| Helman^8^ | focus group | all | Prototype | Understanding the explanation | there was some insight about why the IDSS was making a particular risk prediction | “I like the fact that the fluid responsiveness, arterial tone, cardiac performance provides information that kind of allows you to at least see and judge what data is being used to…drive this. So, you can have a better idea, instead of…the feared black box…” “It might be helpful, with explanations…just like a verbal or a text explanation of…how the systems’ coming to that. Like a little bit more background of how it’s coming to that decision…I would just…want a little bit more background information on why it’s making certain recommendations.” |
| Helman^8^ | focus groups | Clinicians (various) | GUI prototype | Performance (Model) | ensure intelligent decisional support system (IDSS) would capture the heterogeneity of various patient presentations | “You know I think there’s just a very broad scope of pathology that we see and… potential physiologic derangements…, I would just want to feel very confident that this algorithm could accommodate all of those things…” |
| Helman^8^ | focus groups | Clinicians (various) | GUI prototype | Knowledge base | validate the intelligent decisional support system (IDSS) output with clinician assessment findings and cardiorespiratory monitor vital signs. | “…AI [artificial intelligence] algorithms obviously are a function of the input data…but it’s so lovely that we can quickly validate both the heart rate and blood pressure there. So that if the AI [artificial intelligence] seems a little bit off, we can use our own clinical skills to know whether it’s accurate or not…” |
| Helman^8^ | focus groups | Clinicians (various) | GUI prototype | Data quality | the IDSS output is only as valuable and accurate as the data that are put into it |  |
| Helman^8^ | focus groups | Clinicians (various) | GUI prototype | Evidence | desire to see the highest level of evidence/transparency derived from randomized clinical trial results before any meaningful use would be enacted. | “Until I saw it…used in a randomized control trial…to see if it affected patient outcomes in any sort of meaningful way or would just add an additional cost without really adding additional benefit…I would base my feelings off the results of that study.” |
| Helman^8^ | focus groups | Clinicians (various) | GUI prototype | Autonomy | Many participants were displeased with the recommended “Action” section of the initial GUI prototype that was presented to them. They felt that their professional autonomy would be disrupted if they were asked to follow the IDSS “Actions” and not their own clinical judgement. | “I don’t love the part where it says action and it tells you the recommendations. I don’t like anything that…takes the autonomy away from the practitioner, and the caregiver but…allows people to…just take a recommendation without considering how it might impact that particular patient.” |
| Helman^8^ | focus groups | Clinicians (various) | GUI prototype | Perceived usefulness (easier to complete task) | The clinical participants mentioned unforeseen benefits such as: (1) using the GUI for telemedicine purposes and (2) appreciation for the communication of all pertinent data in one place. | “…When we do telemedicine, Tele-ICU [intensive care unit] and we’re not physically at the bedside to have that palpable assessment, maybe we don’t even have…as close of a working relationship with the staff at the bedside and we are not comfortable with the communication…or you have somebody who is less experienced at the bedside. It’s another data point which can be helpful because you’re scrounging for data points at a distance, so I can see it being helpful there.” |
| Helman^8^ | focus groups | Clinicians (various) | GUI prototype | Perceived usefulness (easier to complete task) | The GUI helping newer clinicians to recognize patient deterioration before a crisis event | “For younger nurses, I feel like that’s…a major part of my job is to…avert a crisis, rather than waiting for the crisis to happen, and I feel like that would be really helpful for new nurses.” |
| Helman^8^ | focus groups | Clinicians (various) | GUI prototype | Autonomy | The GUI would hinder the development of critical thinking skills and how novice clinicians may overly rely on the IDSS output without thinking for themselves. | “More novice practitioners might just see the recommendations on the screen and… automatically follow them without necessarily… applying what the recommendation is to the clinical context and…gauging independently whether or not that’s appropriate.” “My concern is and I’m coming at this because I work with a lot of new nurses and developing that critical thinking…if this might not hamper the natural development of critical thinking skills…I personally…would like having this in my back pocket…What am I missing, this is what I’ve done. Okay I’ve gotten through the fluids. I’ve gotten through the vasopressors, what else might I consider. But for a newer nurse they might not even consider that because, well the computer will just tell me.” |
| Helman^8^ | focus groups | Clinicians (various) | GUI prototype | Perceived usefulness (easier to complete task) | For the more advanced clinician, they felt that the GUI could provide the evidence/confidence needed to back up their clinical intuition when patient deterioration is looming while vital sign changes are often delayed in presentation. | “The AI [artificial intelligence] suggestions are only a bonus that it can either help me…feel confident aboutmy impression or…maybe keep me aware if I’m…missing something.” |
| Helman^8^ | focus groups | Clinicians (various) | GUI prototype | Perceived usefulness (decreases time) | use the GUI as a communication tool when summarizing and translating their patient impression to medical team members when escalating a concern, as well as during hand-off and shift change | “I would use it to help communicate with the doctors, interventions that they may not have thought of that we could be doing.”  “So I have a very intimate picture of the patient’s clinical status, but when I need to tell the physician and…relay that information, a lot of times they just want to see the highlights. Okay, what is the problem, and this would be a great way to sort of sumup everything that I’m trying to tell them in one visualization So yes, I think that would also be a very useful tool in communicating with the physicians or the practitioners that we’re working with.” |
| Helman^8^ | focus groups | Clinicians (various) | GUI prototype | Education and training | They observed the length of time that it took to explain how to use the GUI and stated their worries about feasibility in the clinical environment (explanations for use will have to be<5minutes) | “It took…a good five or so minutes just to explain it. I think is…a specific challenge too and then answering questions. So, if people are having issues just understanding what it’s showing and what it means I think that’s going to be a barrier to get people to even start to use it, especially on a regular basis.” |
| Helman^8^ | focus groups | Clinicians (various) | GUI prototype | Perceived usefulness (increases workload) | Concerns about prioritizing their workload; will the GUI create additional work? |  |
| Helman^8^ | focus groups | Clinicians (various) | GUI prototype | Perceived usefulness (decreases time)/ suggestions for improvement | Integration of vital sign data and medical interventions side-by-side in a real-time view. Clinicians commented on the perceived time this design detail could potentially save. | An advantage that could potentially save time, improve workflow, and decrease workload in already demanding clinical environments. “Significantly shorten the amount of time that it takes me to go in and review vital signs, click which ones I want, decide which timeframe within [electronic health record]…I would be very excited to use this just to save time from what is a tedious means of finding and then switching to the MAR [medication administration record].” |
| Helman^8^ | focus groups | Clinicians (various) | GUI prototype | Perceived usefulness (easier to complete task) | They imagined their own clinical decision-making and recognition processes would occur first, as if they already knew patient instability was occurring. The IDSS predictions communicated through the GUI would be used as an adjunct to validate their decisions driven by intuition or their “gut feelings.” | “I don’t think it would change my decision making very much at least not the status part, maybe the forecast. But I’m still going to make the decisions that I’m going to make, especially if I feel…get the gut feeling that something’s not right with my patient. No matter what the monitor says, I’m going to…investigate that.” |
| Helman^8^ | focus groups | Clinicians (various) | GUI prototype | Perceived usefulness (easier to complete task) | in a scenario where the clinician did not know what was driving patient instability or what the appropriate intervention should be, then the IDSS predictions communicated through the GUI would be used as a first pass “consultant” and not validation for decision-making. | “I think I would probably use it as a consultant at a decision node. So, for example, if there was a patient in whom I wasn’t sure what the right thing would be to do. I would probably consult this and, in that, would almost be nice to have.” |
| Helman^8^ | focus groups | Clinicians (various) | GUI prototype | Patient/ carer impact | Clinicians were concerned about how the GUI could encourage family member distrust if the providers or nurses did not follow through with the IDSS recommendations. | “Some hesitancy to have it fully accessible at the bedside with the fact that there’s family members…and you know they’re already watching every single blip.” “My only concern about it being in the room is…it’s another area of stress for the families. It’s making recommendations and you’re not doing what the machine is recommending. To cause some mistrust with the health care providers, but I do like the idea of it being able to be in a separate window, so that you can keep it…minimized and pull it up…” |
| Henry^9^ | Interviews | Physicians and nurses | Post-implementation | Education and Training | clinicians lacked an understanding of how specifically the ML-based system achieved higher reliability than the prior CDSS. While describing the model behind the ML-based system as being generally more “sophisticated” than a conventional CDSS, most could not identify how the internal logic of the ML-based system differed from that of rule-based CDSS, often attributing improvements solely to the fact that the ML-based system considers a larger number of measurements. |  |
| Henry^9^ | Interviews | Physicians and nurses | Post-implementation | Education and Training | Clinicians had an incorrect understanding of the ML-based model, assuming that it (like the prior CDSS) simply checked whether parameters exceeded established thresholds. In actuality, instead of using prespecified thresholds, the ML behind the system learned multidimensional indicators of risk from the data, combining patient history and clinical presentation into a predictive risk score. |  |
| Henry^9^ | Interviews | Physicians and nurses | Post-implementation | impact on workflow | Regardless of their understanding of the ML behind the system, physicians were generally responsive to its alerts and integrated them into their diagnostic process. |  |
| Henry^9^ | Interviews | Physicians and nurses | Post-implementation | Autonomy | they saw themselves as maintaining ultimate responsibility for diagnosis and treatment decisions. Thus, physicians acknowledged that an alert might “make you think of [an] alternative diagnosis” but this was differentiated from “swaying” or “influencing” the physician. |  |
| Henry^9^ | Interviews | Physicians and nurses | Post-implementation | Knowledge base | In cases where they disagreed with the system recommendation (sepsis or not), physicians reported that they would rely on their own judgment. |  |
| Henry^9^ | Interviews | Physicians and nurses | Post-implementation | Knowledge base | Clinicians also differentiated their diagnostic process from the capabilities of ML, emphasizing elements of clinical expertise and intuition that they felt ML could not replicate. Multiple providers referenced the visible cues and richer information available from interacting with the patient at the bedside. | An ED physician expressed, “[The system] can’t help you with what it can’t see.” |
| Henry^9^ | Interviews | Physicians and nurses | Post-implementation | Perceived usefulness (easier to complete task) | Clinicians also found value in the ML-based system beyond the point of diagnosis. Both nurses and physicians reported viewing the system as a “second pair of eyes” to bring cases to their attention or to alert them to a change in the patient’s state. |  |
| Henry^9^ | Interviews | Physicians and nurses | Post-implementation | Perceived usefulness (decreases workload) | This ongoing monitoring by the system was experienced as alleviating some demands on their attention and cognition in a context of feeling “bombarded with clinical information.” | “anything that helps cognitive unloading.” |
| Henry^9^ | Interviews | Physicians and nurses | Post-implementation | Actionability/ Perceived usefulness (easier to complete task/ decreases time) | They additionally described the system as helping to prompt time-dependent actions and to coordinate across multiple care team members. | A nurse described, “I think we try to get them in front of a provider a little bit quicker or get some of the stuff started out in triage.” |
| Henry^9^ | Interviews | Physicians and nurses | Post-implementation | Explanation needs | None of the clinicians fully understood the machine learning behind the system; however, while some were curious to learn more, they did not perceive that understanding the system’s logic in an individual case would change their decision-making. | For clinicians, I think just understanding [that] this is a machine learning tool and it does data mining, I think will be more than enough.” |
| Henry^9^ | Interviews | Physicians and nurses | Post-implementation | Performance (model) | Although unconcerned with the specific statistical model behind the system, clinicians reported having come to better understand how the system operated by observing its behaviour in different scenarios and with different patient types. |  |
| Henry^9^ | Interviews | Physicians and nurses | Post-implementation | Evidence | Clinicians valued external studies of the system and recommendations by colleagues and experts that allowed them to develop trust peripherally. Many clinicians described using the system because a colleague or department head had endorsed it, or they had seen descriptions of the system’s development and validation process. | “I’d want to understand the population it was derived from… and then I’d want to see the population that they validated it on afterwards… whether that group looks like the patients that I’m treating.” |
| Henry^9^ | Interviews | Physicians and nurses | Post-implementation | Development/ Design | Clinicians also valued that they were able to ask questions about system design choices during educational sessions and customize the interface and alert sensitivity to their environment and patient population. | “I need to understand the motivation behind that tool because when I apply that tool, I’m applying the judgment of the creators of that tool.” |
| Henry^9^ | Interviews | Physicians and nurses | Post-implementation | Development | Interacting with the deployment team also allowed for input into the tool’s operations, which was described as an improvement over prior CDSS deployments. |  |
| Henry^9^ | Interviews | Physicians and nurses | Post-implementation | Autonomy | Several pointed to the potential for over-reliance on automated systems, which could ultimately degrade their clinical abilities | “I think [that] there are a lot of people, frankly, that will quickly default to having a tool tell them what to do and stop assessing, and I hope that’s not true, but I’ve seen it happen.” |
| Henry^9^ | Interviews | Physicians and nurses | Post-implementation | Autonomy/ Patient/carer impact | Several also mentioned concerns that regulatory agencies might use these systems to standardize care even in scenarios where a clinician disagrees with the system, potentially leading to overtreatment and patient harm, especially in cases where the alerts occurred prior to clinical recognition. |  |
| Henry^9^ | Interviews | Physicians and nurses | Post-implementation | Suggestion for improvement | When asked what would convince them to act on the system’s recommendations prior to apparent symptoms, suggestions included clinical trial evidence, or personally experiencing scenarios where the alert was dismissed but the patient was later diagnosed as having sepsis. |  |
| Jauk^10^ | Focus group | All (physicians and nurses) | Post-implementation | Perceived usefulness (easier to complete task) | Application is useful support for early recognition of delirium | “The application gives good support – I am convinced of its usefulness.” “Due to the delirium prediction application, we were already able to prevent the sliding into a strong delirium with simple interventions.” |
| Jauk^10^ | Focus group | All (physicians and nurses) | Post-implementation | Perceived usefulness (decreases workload) | Application helped to reduce resources for screening | “I see the application as a benefit, as we are able to reduce the time for delirium screening.” |
| Jauk^10^ | Focus group | All (physicians and nurses) | Post-implementation | Perceived usefulness (easier to complete task) | Application provided support in the assessment of patients under sedation at admission | “It is especially an added value if patients are not responsive during admission.” |
| Jauk^10^ | Focus group | All (physicians and nurses) | Post-implementation | Perceived usefulness (easier to complete task) | Application was used to confirm existing presumptions on delirium risk | “The prediction helps to corroborate my own estimation when seeing a patient.” “Also, the prediction helps us when we are not quite sure about the delirium risk.” |
| Jauk^10^ | Focus group | All (physicians and nurses) | Post-implementation | Perceived usefulness (easier to complete task) | Support targeting of patients with a delirium diagnosis in a previous stay | “Especially patients with a diagnosis of delirium in the past are being targeted earlier now.” |
| Jauk^10^ | Focus group | All (physicians and nurses) | Post-implementation | Design | Appreciation for no need of additional data entry |  |
| Jauk^10^ | Focus group | All (physicians and nurses) | Post-implementation | Impact on workflow | Prediction was available within few seconds in the user interface of the health information system |  |
| Jauk^10^ | Focus group | All (physicians and nurses) | Post-implementation | Design | The visualization in the web application sparked much enthusiasm, because it provided a comprehensive view of a patient supporting healthcare quality not connected to delirium prevention. |  |
| Jauk^10^ | Focus group | All (physicians and nurses) | Post-implementation | Performance (model) | the predictive accuracy of the algorithm was perceived as very high. | “The system has almost 100 % accuracy.” “There are not too many patients in the very high risk group – it seems correct.” |
| Jin^11^ | interview | physicians | high fidelity prototype 1 | Understanding the explanation | Support for identifying contributing factors in predictions: One common demand proposed by the physicians is to make the prediction result easier to interpret. | “We sometimes do not trust the predictions generated by machines, especially when [they disagree] with our judgement. It would make a difference if the model [could] tell us the reason [for] the prediction.” |
| Jin^11^ | case study | pulmonologist | high fidelity prototype 2 | Understanding the explanation | the progression of the similar patients provided more information to confirm the prediction results via the summarization of the raw data. | “It can inspire me to make better decisions. We can also learn from the progression of similar patients.”; “The progression of the similar patients confirmed that heparin sodium can lower the risk of obstructive chronic bronchitis. The result actually reminded me of a new research work in medical science. This research work evaluated the therapeutic use of heparin in patients with COPD (chronic obstructive pulmonary disease)” [ |
| Jin^11^ | case study | pulmonologist | high fidelity prototype 2 | Actionability | Outcome analysis task was seen as useful. (Doctor removed all of the events for heparin sodium from the sequence, resulting in a significantly increased risk of pulmonary diseases) | “This function is really novel and useful. Knowing the effect of the treatment in advance can be very helpful in clinical scenarios. Providing the outcome analysis result can also make the patients more confident in making treatment plans.” |
| Jin^11^ | interview + demo | 2 physicians | high fidelity prototype 1 | Perceived usefulness (easier to complete task) | Support for predicting the risks of potential diseases: predicting the risks of potential diseases based on historical record is useful in verifying their initial diagnosis. I.e. The system should be able to automatically assess a patient’s historical medical record to predict the risks of a set of potential diseases identified by the physicians | “I’ll confirm the diagnoses with high risk and make further investigation to assess those with low risk.” ; “I’ll be more confident in making decisions if the prediction is aligned with my judgement.” |
| Jin^11^ | interview + demo | 2 physicians | high fidelity prototype 1 | Explanation needs | Support for exploring predictions under the context of historical sequences: important to review predictions with historical records. The system should be able to illustrate the prediction results within the context of the patient's medical historical medial record to facilitate data exploration and result interpretation | Historical events can provide evidence for us to determine whether the prediction is trustworthy,”  “Reviewing history sequence can help us better understand the predictions.” |
| Jin^11^ | interview + demo | 2 physicians | high fidelity prototype 1 | Perceived usefulness (easier to complete task) | Support for easy comparison between the focal patient and similar patients: The physicians expressed their need for leveraging the past experience in diagnosing and treating patients with similar clinical pathways. | “Knowing how patients with similar symptoms [were] diagnosed and treated before will provide us with more guidance in making [a] diagnosis and treatment plans.” |
| Jin^11^ | interview + demo | 2 physicians | high fidelity prototype 1 | Explanation needs | Support for exploring the outcomes of possible treatment plans: When discussing the requirements for supporting the prognosis, P1 expressed a desire for simulating possible treatment plans | “We usually have multiple treatment plans in mind. It would be great if the tool [could] help us predict and compare the outcomes after [making] those treatment plans.” “It can also help us understand the effect of the drugs on the disease through the exploration.” |
| Jin^11^ | case study (think aloud) | cardiovascular doctor | high fidelity prototype 2 (evaluation) | Explanation needs | Viewing similar patients and their disease progression paths gave him more confidence in the prediction results as “it provides specific evidence [to support the prediction results].” | “it provides specific evidence [to support the prediction results].” |
| Jin^11^ | case study (think aloud) | cardiovascular doctor | high fidelity prototype 2 (evaluation) | Perceived usefulness (easier to complete task) | similar patient view would useful for medical researchers | useful for medical researchers as “it illustrates many examples following different treatment plans |
| Jin^11^ | case study (think aloud) | cardiovascular doctor | high fidelity prototype 2 (evaluation) | Knowledge base | Removing or adding features. The correctness of the various predictions were verified by the doctor |  |
| Jin^11^ | interview and FG (+ demo and system use) | physicians | high fidelity prototype 2 (evaluation) | Performance (model) | the capability of the system in estimating risk of potential diseases is useful | “I need to take care of over 50 patients a day . . . sometimes I am just too tired to avoid making mistakes . . . if the system is developed based on statistical analysis of similar medical records, I’d love to trust the results . . . and it can actually help us reduce the risk of making a mistake.” |
| Jin^11^ | interview and FG (+ demo and system use) | physicians | high fidelity prototype 2 (evaluation) | Perceived usefulness (easier to complete task) | the system could help inexperienced doctors or medical students make more accurate diagnoses. |  |
| Jin^11^ | interview and FG (+ demo and system use) | physicians | high fidelity prototype 2 (evaluation) | Perceived usefulness (decreases time) | system can help doctors make diagnoses more efficiently, and they felt that the system “can be applied to assist consultation.” | “This tool can [help] reduce a doctor’s burden.”  “Doctors’ time is valuable, [and] quickly estimating the risk of a patient [using the system] is a useful function.” |
| Jin^11^ | interview and FG (+ demo and system use) | physicians | high fidelity prototype 2 (evaluation) | Knowledge base | comparing the focal patient to similar patients “accords with the idea of evidence-based medicine.” | “We used PSM to study the effect of clinical decisions based on statistical analysis. However, it is generally hard to find [a] proper study group [due to the high complexity of observational data] . . . it is very powerful that your tool can automatically identify patients with similar progression and make comparison with rich contextual information.”; “Comparing to the similar patients in detail not only gives me more confidence of the prediction results but also provides me with rich treatment examples.” |
| Jin^11^ | interview and FG (+ demo and system use) | physicians | high fidelity prototype 2 (evaluation) | Perceived usefulness (easier to complete task) | [Treatment outcome analysis] Participants felt that the idea of virtually making different care plans and comparing their potential outcomes was a “cool and valuable” feature to support making a prognosis | “this system provided an interactive way for exploring some complicated situations and their influence on the patient.”  “We used to study the effect of a single treatment with [a] RCT (randomized clinical trial), [whereas] this tool can simulate the effect of a combination of treatment, which is awesome.” |
| Jin^11^ | interview and FG (+ demo and system use) | physicians | high fidelity prototype 2 (evaluation) | Perceived usefulness (easier to complete task) | Knowing the impact of treatment on the risk of diseases is convenient for doctors to further investigate key factors that influences the outcome | “this can be used to help junior doctors try out the dose of insulin in treating diabetics.” E5 agreed and added, “This system can help junior doctors learn from the experience of senior doctors in a more interactive way instead of plain text [in] the textbook.” |
| Jin^11^ | interview and FG (+ demo and system use) | physicians | high fidelity prototype 2 (evaluation) | Design | system was more complex than any tools they had used in their daily work. | “looks overwhelming” and “seems difficult to learn” |
| Jin^11^ | interview and FG (+ demo and system use) | physicians | high fidelity prototype 2 (evaluation) | Education and training | after they became familiar with the system’s functionalities and explored the system by themselves for a while, they felt that the design was “intuitive” and the usage of system “gradually becomes clear.” once they became familiar with the design, they felt that this view was “informative” and “clearly illustrated different care plans and the corresponding outcomes.” | “I was confused by so many views and had no idea where to look at, but then I realized that each view is especially designed for a specific task, and that [what analytical task to perform] is all I need to care about.” E7 agreed and added “especially that the [exploration] pipeline for each task is relatively fixed.” E5 felt that the interaction “helped a lot in reducing the complexity,” as he explained while tweaking the treatment plan: “Tools we used typically employ a lot of context-menu [to interact with users], which is difficult tomemorize, [whereas] this tool allows me to manipulate the visualization more directly.” |
| Jin^11^ | interview and FG (+ demo and system use) | physicians | high fidelity prototype 2 (evaluation) | Education and training | the difficulty [reading statistical diagrams and charts] mainly comes from the lack of training | “We (doctors) spend years in school to learn how to make [a] diagnosis based on those [traditional] statistical tools and diagrams . . . your tool is obviously more informative but we just need more time to get familiar with it.” |
| Jin^11^ | interview and FG (+ demo and system use) | physicians | high fidelity prototype 2 (evaluation) | Education and training | agreed that they can use the system easily after training. | “It will take some time for us to learn how to use the system. However, the design is not difficult to understand. After the training, I am willing to use the system. I also hope to get some new findings using this system.” |
| Jin^11^ | interview and FG (+ demo and system use) | physicians | high fidelity prototype 2 (evaluation) | Data quality | concerned about the quality of the training data that directly influenced our analysis results. | that the quality of the EHRs collected in Chinese hospitals were much worse than those of the MIMIC dataset. For this reason, they believed that CarePre might not be as useful in Chinese hospitals right away. |
| Jin^11^ | interview and FG (+ demo and system use) | physicians | high fidelity prototype 2 (evaluation) | Data quality | prognosis estimate was useful only when the underlying data were rich enough to represent the rich variety of outcomes that patients face | E2 reminded us that a prognosis estimate is usually based on the statistics of a very large collection of patients over a very long period of time |
| Jin^11^ | interview and FG (+ demo and system use) | physicians | high fidelity prototype 2 (evaluation) | Data quality | the data used in the system need to be updated as well | E4 added that the knowledge in the field of medicine updated very fast. |
| Jin^11^ | interview and FG (+ demo and system use) | physicians | high fidelity prototype 2 (evaluation) | Design | although CarePre was useful, the design was not sufficient, as it has limited ways for clinicians to enter new medical data. | When compared to the existing system, your tool focuses more on the analysis but lacks a convenient method for me to enter medical records in the text form.” |
| Matthiesen^12^ | walkthrough | cardiologists | near-live | Understanding the explanation | presentation of important parameters provided explainability and supported decision-making by resembling the clinical interpretation process of what counts for or against the occurrence of VT/VF | "To list what counts for and what counts against, makes really good sense. That’s also how it works in my head" |
| Matthiesen^12^ | walkthrough | cardiologists | near-live | Explanation needs | Listing the algorithm’s important parameters enabled electrophysiologists to do in-situ validations of the prediction tool’s predictions by interpreting the data against the patient case | "It's very nice to see that the algorithm reacts on the same parameters that I've discovered myself ... So it's nice to see that I agree with it. You could say that it’s supporting and it's safe to know, that it also says there was something here." |
| Matthiesen^12^ | interview | cardiologists | near-live | Knowledge base | Visualization of important parameters created more confidence in the prediction tool than the probability score alone as the tool summed up many of the same assumptions that the electrophysiologists already had | "I think it's super good, I actually think it's really pedagogical, I like it. Because, in reality this is how it confirms the result. It’s basically the same empirical data that you have in your mind: You say “okay, is this a case where we have to do something?” It sums up some assumptions that you have made yourself, and in that way, I actually think you are confirmed more than if you have a green or red light." |
| Matthiesen^12^ | walkthrough | cardiologists | near-live | Knowledge base | Presentation of important parameters provided explainability and supported decision-making by resembling the clinical interpretation process of what counts for or against the occurrence of VT/VF | "To list what counts for and what counts against, makes really good sense. That’s also how it works in my head" |
| Matthiesen^12^ |  | cardiologists | near-live | Perceived usefulness (easier to complete task) | The prediction tool was helpful and increased their confidence in their choice of clinical action | "Well, it hasn’t changed my current decision, but the basis is much better, and I can easily see that it has helped me." |
| Matthiesen^12^ | interview | cardiologists | near-live | Perceived usefulness (easier to complete task) | The predictions could help prioritize patients | "If you are in a busy situation where many transmissions have arrived and the technician and I have to maneuver and prioritize, there is no doubt that we will concentrate on those with high-risk predictions." |
| Matthiesen^12^ | walkthrough | cardiologists | near-live | Impact on workflow | The predictions could determine what action to take in relation to the local circumstances at the clinic | This [tool prediction] is something that might make me react a little more aggressively. […] Now I've been told that he's actually more likely to get an episode within the next month than he's not getting an episode […] if our program is fully booked, both today and tomorrow, and the day after tomorrow, but on Friday we have a time. Then I kind of have to make a trade off if I really want to spare him a shock. Which may turn into a lot of shocks |
| Matthiesen^12^ | walkthrough | cardiologists | near-live | Perceived usefulness (easier to complete task) | The prediction tool was found to assist decision-making by confirming the electrophysiologists 'clinical evaluations and expectations of an increasing risk of VF/VT | "So, I agree with the conclusion, it was also my feeling that I would be a little worried about this patient." |
| Matthiesen^12^ | walkthrough | cardiologists | near-live | Perceived usefulness (harder to complete task) | When the electrophysiologists were focused on predicting arrhythmias other than VT/VF, the prediction tool was deemed less useful, |  |
| Matthiesen^12^ | walkthrough | cardiologists | near-live | Perceived usefulness (easier to complete task) | The predictions served as a second opinion | "But then if it is you now have to convince some [other electrophysiologists] that they should ablate her, then instead of saying that I think so, you can argue that the algorithm thinks so too. So, in that way you can say that you can get an extra view of it." |
| Matthiesen^12^ | walkthrough | cardiologists | near-live | Perceived usefulness (easier to complete task) | The prediction tool was helpful for collecting arguments that supported the electrophysiologists when trying to "gather thoughts" about potential VT/VF occurrences | "In that way, the algorithm can be a support because it helps to gather thoughts about things that play a role in whether a person gets a new arrhythmia." |
| Matthiesen^12^ | walkthrough | cardiologists | near-live | Performance (model) / Data quality | In some cases, the electrophysiologists found that the parameters were based on wrong data from the ICD transmission. In those cases, it enabled electrophysiologists to check if the prediction tool based its predictions on wrong or poor data quality and to decide whether to trust the predictions or not. | "What's happening here is that the [system] detects that the patient has VT, and then the prediction tool bases it’s predictions on that. But it’s not entirely correct, because the device has recently been re-programmed to sense everything" |
| Matthiesen^12^ | walkthrough | cardiologists | near-live | Knowledge base | Listing the algorithm’s important parameters enabled electrophysiologists to do in-situ validations of the prediction tool’s predictions by interpreting the data against the patient case | "It's very nice to see that the algorithm reacts on the same parameters that I've discovered myself ... So it's nice to see that I agree with it. You could say that it’s supporting and it's safe to know, that it also says there was something here." |
| Matthiesen^12^ | walkthrough | cardiologists | near-live | Perceived usefulness (easier to complete task) (decreases workload) | Once they become familiar with the system, they expect the AI tool will speed up decision-making and reduce the diagnostic workload | "It will give me a much better basis for decision making and I actually think it will save me a lot of time. Just like with all other new technology based on machine learning: the first 2 months I sit and read through to see what I have, but in month 3, I will look at the output alone. Because then I trust that it has pulled out what is appropriate, and then it starts saving me all the work I did in the beginning. But for everyone, it is that there is a phase for you personally to find out if this brings you further. […] I really think I would have come to the decision faster if I had seen this first." |
| Matthiesen^12^ | walkthrough | cardiologists | near-live | Perceived usefulness (decreases time) | Time can be saved when personal trust in the prediction tool is developed | It will give me a much better basis for decision making and I actually think it will save me a lot of time. Just like with all other new technology based on machine learning: the first 2 months I sit and read through to see what I have, but in month 3, I will look at the output alone. Because then I trust that it has pulled out what is appropriate, and then it starts saving me all the work I did in the beginning. But for everyone, it is that there is a phase for you personally to find out if this brings you further. […] I really think I would have come to the decision faster if I had seen this first. |
| Matthiesen^12^ | walkthrough | cardiologists | near-live | Perceived usefulness (easier to complete task) | The tool could help support decision-making when patient input is inaccessible | "I might reach a decision faster with this system if I can’t get a hold of the patient i.e., if the patient does not pick up the phone. Then it could well be that I look at the alarm and say “well, yes okay there is low risk.”" |
| Matthiesen^12^ | walkthrough | cardiologists | near-live | impact on workflow/ perceived usefulness (decreases workload) (decreases time) | Tool can support workflow and reduce unnecessary time consumption for electrophysiologists by delegating decision-making to the technician |  |
| Matthiesen^12^ | walkthrough | cardiologists | near-live | Performance (model) | Acceptability of the prediction tool was high when patient cases concerned VT/VF, as the risk predictions were found to be relevant. |  |
| Matthiesen^12^ | interview | cardiologists | near-live | Perceived usefulness (easier to complete task) | expectations that the prediction tool would bring new and groundbreaking insights to support or challenge their decisions on which action to take /// Nice to have rather than need to have | "[…] it confirms the assessment you make, and that's fine, but it's not something groundbreaking, and that's okay too." |
| Matthiesen^12^ | interview | cardiologists | near-live | Performance (model) | high precision is important for prediction tool adoption to happen; false positives or negatives hinder adoption | "It needs to be easily accessible and we [team of electrophysiologists] have to agree that we trust it [the prediction tool]. We just have to say that yes it looks right. For example, the Optivol alarm had too many false positives, which gave a lot of extra work and everything, and we actually chose not to use it because there were too many sources of error, and you only really discover that when you work with it [new algorithms]." |
| Matthiesen^12^ | interview | cardiologists | near-live | Impact on workflow | acceptance and clinical adoption are collectively decided based on team experiences from real-world use | "I would say that it [prediction tool] would be an instrument that would have to be accepted in our group and then you would find it valuable when we all agree to take the red alarms first, and in that way use it to prioritize a bit." |
| Matthiesen^12^ | interview | cardiologists | near-live | Performance (model) | acceptance and clinical adoption are collectively decided based on experiencing that the prediction tool actually confirms decisions in everyday clinical practice | "I just think I should see that it confirms our decisions in enough cases - then I would feel comfortable about colleagues leaning on it […] There is something about trying it out, you know how it is." |
| Matthiesen^12^ | interview | cardiologists | near-live | Evidence | Adoption can also be achieved through building trust in the tool by means of validation studies. trust is a precondition for adoption, which can be achieved by documenting effects in a randomized clinical trial and through algorithm validation in peer-reviewed journals | "Published studies of the algorithm would increase confidence yes, because then you know that someone with an understanding of making these models have said that it looks okay; someone externally who have validated it." |
| Matthiesen^12^ | interview | cardiologists | near-live | Perceived usefulness (easier to complete task) (decreases time) | Prediction tool useful for supporting more efficient prioritization and identification of important patient cases | "When transmissions come in, it’s almost an unsorted list of transmissions […] The list is unprocessed, so with the algorithm it takes it a step further by nuancing what comes into CareLink [Medtronic’s remote monitoring dashboard] with some semi-quantitative markings. And, if it is reliable, then it would be valuable. Partly because you don’t overlook anything, and partly because you are confirmed that we must take these patients first, because we have experience that there can be trouble here." |
| Matthiesen^12^ | interview | cardiologists | near-live | Autonomy | The prediction tool could function as “data help” by enabling junior doctors to get a form of senior help by consulting the tool | "You could say that in this way, the young doctor can do without getting senior help by actually getting data help." |
| Matthiesen^12^ | interview | cardiologists | near-live | Design | The intention of using the prediction tool is dependent on easy access, as well as how well it presents data and alleviates the need for clicking through several web pages in remote monitoring systems | "If it’s easily presented and you don’t have to go in and look through 4 pages and such and if it was on the front page and brought up “number of episodes” and information like that - if you could easily retrieve the information [from the prediction tool] or if it was printed on the list of transmissions we are working on, then it would also be a great help." |
| Matthiesen^12^ | interview | cardiologists | near-live | Data quality | It is practical that the algorithm uses data already available in remote monitoring systems, which are used daily for decision-making in the clinic. Knowing the data creates transparency and enables in-situ validation of the correctness of the probability score, thereby increasing the likelihood for success with implementation of the prediction tool | "What one would emphasize, is that the algorithm uses the same data that the clinician uses i.e. it’s the same data, just integrated according to a formula that clinicians do not currently have available" |
| Pinto^13^ | interview | Clinicians | prototype | Knowledge base | Clinicians did not trust a particular feature based on EEG data as this data is often inaccurate (based on artifacts) | Clinicians found this suspicious as scalp EEG does not fully capture gamma rhythms, and thus, this predominance of gamma features might be explained by the presence of artifacts. |
| Pinto^13^ | interview | Clinicians | prototype | Knowledge base | Explanations that make sense provided higher trust | Curiously, topological/anatomical explanations of the chosen electrodes were not particularly fundamental compared with features. |
| Pinto^13^ | interview | data scientists and clinicians | prototype | Explanation needs | Concepts of medical knowledge differs between data scientists and clinicians | Clinicians often consider the preictal period a fast spontaneous phenomenon that might start some seconds before the seizure onset. Data scientists' strategy is different: They aim to capture a slow transition from a background state into a seizure, by classifying consecutive windows of EEG as preictal/interictal. These notions of seizure generation are different. |
| Pinto^13^ | interview | Clinicians | prototype | Explanation needs | If clinicians cannot explain pre-seizure mechanisms several minutes (or even hours) before seizure onset, data scientists will not be the ones doing it as they cannot provide a clinician-comprehensible answer | “until proven otherwise, everything is an artefact.” |
| Pinto^13^ | interview | Clinicians | prototype | Performance (model) | Their curiosity to understand how our models would behave when patients performed daily activities. if we could guarantee that our models would not confound these activities with a pre-seizure state, we would gain more trust |  |
| Pinto^13^ | interview | Clinicians | prototype | Performance (model) | Asked about a performance bias toward patients whose training and testing seizures occurred around similar times of the day. |  |
| Samhammer^14^ | interview | clinicians | Pre-implementation | Suggestion for improvement | Suggestion made to implement mechanisms for users to influence which parameters are included in the analysis of the AI-DSS. The physicians want the system to provide traceable prediction. | "I think it's important that you can yourself let your prioritisation flow into it, that it's possible for you, that it can also be changed. That you don't somehow get a score where you had no possibility to influence it. That one simply makes use of the great computing power and also the better concentration ability of many variables, but says oneself: the variable is most important to me, then this one comes, then this one comes. And that you can possibly also say: okay, the result surprises me. Now I turn these two variables around again, because they are almost equally important" |
| Samhammer^14^ | interview | clinicians | Pre-implementation | Evidence | Decisions should be evidence-based (For the sake of patient safety, any chosen treatment must rely on prospective clinical studies which validate its effectiveness) | "So in general I need a study that shows me that the tool brings added value, yes? For example, that it can prevent certain events or make the handling more effective. That it can better predict the critical event, faster, more autonomously. So it must/ needs practically, one study that brings me the proof of effectiveness. Then I start to believe. Yes, and actually itʼs not just one study that is needed, it takes several studies. It needs multicentre systems established in other clinics. Then we start to believe that it will be of some use. Yes, that's just the way it is in medicine" "Yes, […] if it predicts a probability of one hundred per cent and you have tested it in a study beforehand and it always came true one hundred per cent then at some point the trust is there. But/ Yes, you need a study beforehand that proves that. So you need proof that you can get involved in a black box" |
| Samhammer^14^ | interview | clinicians | Pre-implementation | Knowledge base | The idea of evidence-based medicine cannot always be attained in everyday clinical practice, e.g., because of imperfect available evidence | "[…] I understand that some things are not always causally explainable. Or (..) whatever I believe, for, well, physicians, it is always nice to have a causal explanation, so that you can somehow imagine it pathophysiologically. But okay, we know that association is not causality. That is why a pure association, which is not causal, would also be/ It is the case with many things that they are associated but not causally linked, so that it is probably not always possible and I would also be satisfied if I knew there was a, well, coincidence, or something like that. But this one may not be causal, but it still helps me in predicting infections, or something like that" |
| Samhammer^14^ | interview | clinicians | Pre-implementation | Impact on workflow | Physicians describe a need to incorporate AI-DSS into existing decision-making processes. |  |
| Samhammer^14^ | interview | clinicians | Pre-implementation | Explanation needs | Using AI-DSS does not absolve the physicians from explaining the results of the system to the patient. At this point a need for transparency and explainability becomes apparent. |  |
| Samhammer^14^ | interview | clinicians | Pre-implementation | Perceived usefulness (easier to complete task) | Objective facts are used for diagnosis. It creates the image of another person in the room which has the special ability to elevate objectivity | "That something objective is added. So that there is a lot of interaction with the patient and subjective assessments. And that one/that such a system, yes, like [.] an additional objective further arm, as if someone sits beside one" |
| Samhammer^14^ | interview | clinicians | Pre-implementation | perceived usefulness (easier to complete task) | AI-DSS supports need to receive confirmation and support in decision-making or to have an AI-DSS as an additional safety net | "[I]f AI can do a better job, whether someone is compliant or not, and whether it significantly increases the risk. Then it would also be a point to intervene earlier or to say: Look here, my artificial intelligence is warning me, or tells me that this is very strongly increased in the next few days, months. Maybe like having a whisperer, yes, who helps you with the decisions" |
| Samhammer^14^ | interview | clinicians | Pre-implementation | Autonomy | It is assumed that the use of an AI-DSS will lead to a more critical attitude toward one’s own decision-making, | "I think in the end I would also be quite sceptical, but probably I would of course trust my thoughts also then, right? If I can't understand why such a programme can tell me something, I can't use it. But if it were on paper, of course I would have to think very critically and see where I might have overlooked something myself" |
| Samhammer^14^ | interview | clinicians | Pre-implementation | Autonomy | For others the danger of feeling confirmed too quickly is highlighted. While the usage of an AI-DSS can lead to ease in the decision-making process, it could prevent the development of a clinical “instinct.” especially less experienced physicians should be able to make their decisions without additional help and thus gain important experience. | "I think the concern is always a little bit that also young colleagues quickly fall back on artificial intelligence and don’t shape their own instinct that much. And so that’s why I think/So for me, I would do it in a way that, yeah, I see that as confirmation or incentive or further input but try to stay with myself in this whole decision-making process. It’s a nice, yeah, on-top thing, but shouldn’t become the base, I think" |
| Samhammer^14^ | interview | clinicians | Pre-implementation | Patient / carer impact | Physicians must still be able to justify why a particular system is used and explain how it arrives at its result to patients in a way they can understand | "I think it would be more difficult if you can't understand the decisions of the system, right? So, somehow the system has to be very transparent and has to list exactly what the decision it is making or the prediction it is making is based on. Because at the end of the day, it's not the system that talks to the patient, at least not yet, but we as physicians. And then I think we should know what these decisions are based on, so that we can communicate them openly and clearly" |
| Samhammer^14^ | interview | clinicians | Pre-implementation | Explanation needs | An AI-DSS should always have a certain degree of transparency | "I still think that I would not use a system that I had to trust to such an extent that I could no longer understand the decision myself" |
| Samhammer^14^ | interview | clinicians | Pre-implementation | Explanation needs | Physicians show awareness that it is a characteristic of many relevant forms of AI that not every step of the analysis can be made completely comprehensible | "I think the black box is totally okay, because I think I understood that the black box arrived at its results through an extremely large amount of data" |
| Samhammer^14^ | interview | clinicians | Pre-implementation | Explanation needs | System outputs should be reasonably explainable to the treating physician |  |
| Samhammer^14^ | interview | clinicians | Pre-implementation | Evidence | Prospective clinical studies should ensure that the system’s data evaluation is not biased against certain groups of people | "A prospective study would of course also be important again in terms of validation. Or actually to test it again on a larger scale, where there are no biases in it" |
| Samhammer^14^ | interview | clinicians | Pre-implementation | Performance (model)/  Evidence | A system still needs to prove reliable and must be implemented gradually in everyday clinical practice | "For artificial intelligence, I think you have to get used to it a little bit before you also can say this is how it's going to be. Especially in this area, right?" |
| Samhammer^14^ | interview | clinicians | Pre-implementation | Patient / carer impact | Even if a system is based on sound evidence, this does not absolve the treating physicians from their communicative tasks in the physician-patient relationship |  |
| Samhammer^14^ | interview | clinicians | Pre-implementation | Impact on workflow | Some physicians took the given recommendations as a departure point from which to arrive at their own judgment. Nevertheless, a large proportion of physicians preferred to first make their own assessment in its entirety and then use the system’s recommendation to critically question it. |  |
| Samhammer^14^ | interview | clinicians | Pre-implementation | Explanation needs | When the rationale for the recommendation of the system could no longer be understood, a distancing took place. In these cases, the physicians often reverted to their own assessment. | "So if I now, so if I was of the same opinion then I went along with it a bit, but if it was, completely, if I found it completely absurd, then I simply ignored it" |
| Samhammer^14^ | interview | clinicians | Pre-implementation | Evidence | It would be inappropriate to simply accept the recommendation of an AI-DSS, at least not as long as such a system is new, i.e., has not yet proven reliable in trials and practice. |  |
| Samhammer^14^ | interview | clinicians | Pre-implementation | Perceived usefulness (decreases workload) | The reduction of the workload that the use of the system has brought is emphasized. |  |
| Samhammer^14^ | interview | clinicians | Pre-implementation | Autonomy | Emphasizes the concern of becoming “lazy in thinking” if an AI tool is consulted prior to her own approach to the specific case. This suggests that there is a certain reservation about making decision-making too easy in advance. | "I think I always tried to look first myself and then to ask the AI again whether I had overlooked any of the points that the model found important and so on. And have quasi-tried to get an unbiased view on it first and then again, again to let me support so to speak, because otherwise one becomes so lazy in thinking" |
| Samhammer^14^ | interview | clinicians | Pre-implementation | Knowledge base | The willingness to accept the result of the system also depends on the extent to which it coincides with one’s own thoughts about the specific case. Confirmation, for example, led to a reduction of distrust, whereas differences with the system triggered uncertainty. In most cases, uncertainties were used to critically question one’s own assessment | "And actually, it was often reflected what the system gave me and what I somehow found out. I would also claim that it was in the course that I was less suspicious […]. At the beginning like this: Okay, what is this? And then first of all this familiarisation phase with the colour codes and the score. And it has then also always changed a little bit. But my considerations actually were confirmed after the first few times […]. Then I took the information with me, then went in search of it and quickly found myself confirmed" "There was one case where I was annoyed afterwards that I didn't look at the proteinuria, because that was a big point in the AI for this case. And I thought to myself, okay, that's actually something that should have been looked at" |
| Samhammer^14^ | interview | clinicians | Pre-implementation | Autonomy | did not think that by using the system, they would hand over decisional authority to it | […] I think it's just, as I said before, an additional point that, as I said earlier, in this relatively quick and intuitive process, throws a moment of thought in between, even more when you might be in danger of overlooking something. But I think the decision-making process itself is relatively little influenced by that |
| Samhammer^14^ | interview | clinicians | Pre-implementation | Autonomy | The responsibility for making decisions with an AI-DSS is still considered to be with the treating physicia; They suspect that not taking such responsibility for the decision of an AI-DSS could lead to possible diffusions or gaps where no one can be held accountable for an AI-supported decision anymore | "[W]ell, that's also one of the dangers of it, that there is such a diffusion of responsibility in many things, right? […] And with the AI in particular, no one is to blame, because it has been trained with something. And nobody, that's just the way it is, there isn't even a human being who is responsible for the AI now. And in this respect I think it's very important that in the end it's still the physicians decision that stands at the end and is therefore also the one who bears the responsibility. And because an AI can't bear any responsibility" |
| Samhammer^14^ | interview | clinicians | Pre-implementation | Autonomy | handing over responsibility is associated with a loss of authority | "Of course, it would be a problem if I were to make a decision and the patient knew about the decision of the computer programme or the artificial intelligence and it was different from what I would recommend. I think that would be a problem" "Of course, I wouldn't give the patients the feeling that I just give a few values into the computer and their therapy is completely decided by an artificial intelligence programme. But that it is an auxiliary instrument that I, as a human being, overlook as little as possible" |
| Samhammer^14^ | interview | clinicians | Pre-implementation | Perceived usefulness (decreases workload) | such a system could help to carry the heavy burden of responsibility more easily | […] but you could perhaps shift them a bit emotionally. [...] And that would perhaps make life easier for the everyday work of a physician, because quite a few decisions can be depressing [59, senior] |
| Samhammer^14^ | interview | clinicians | Pre-implementation | Perceived usefulness (easier to complete task) | AI-DSS can also be used in the future to develop a more critical attitude in the decision-making process | "So I think that is also a good process that, if you make a deviating recommendation now or come to a deviating result, that you just once again go on the way to look: Did I miss something? And I think that exactly is part of it" |
| Samhammer^14^ | interview | clinicians | Pre-implementation | Perceived usefulness (easier to complete task) | It should prevent wrong decisions but not pre-empt decision making | "I would want to set it up as a kind of prevention system, so that when something happens or something like that, you have this increased awareness or something, but would I integrate it into the direct decision-making process? I don’t know" |
| Samhammer^14^ | interview | clinicians | Pre-implementation | Autonomy | It must be prevented that AI-DSS stands in the way of the acquisition of expertise | "But if you say the AI is not supportive, it is already binding, then it becomes difficult, because maybe my therapy idea is different, so the artificial dictates. Then, I think it actually becomes difficult, because at some point you also come to the point where you simply no longer think sufficiently well about various things yourself. And then you quickly acquire this expert knowledge, which we all try to acquire somehow" |
| Samhammer^14^ | interview | clinicians | Pre-implementation | Education and training | Physicians want more information and further influence on the system. |  |
| Samhammer^14^ | interview | clinicians | Pre-implementation | Impact on workflow / Suggestion for improvement | The system’s assessment should not stand alone, but should be able to be linked to other programs | I think if this AI had also been included [more choices] in the inside, then I would imagine it in the clinical setting. Namely, that I have to access another programme and so on. Now it seems very separate [68, junior] |
| Samhammer^14^ | interview | clinicians | Pre-implementation | Explanation needs | Several physicians would like additional explanations of how the system arrives at its assessment | "For some of them maybe just an explanation why this parameter has maybe an influence, a bit more detailed explanation. Yes, most of the things I could somehow think of [why they are] important for completion or important for rejection, but there were a few where I didn't really understand the direct connection. So maybe a little bit more introduction to the individual ones" "Yes, it would be good to know how it works, what [it] is based on, how it came about in the first place and on which factors. And that it is also explained on the pages that are now available for the cases, whereby for me, who has not yet dealt with it so much, it is still not easy. I also don't know how well one must understand it, but to understand what this [score] or this assessment is based on, that is quite difficult for me to understand. It probably makes sense to give more text and explanation or explanatory models or examples" |
| Samhammer^14^ | interview | clinicians | Pre-implementation | Knowledge base/ Explanation needs | Suggestion that the system should highlight further literature or research results to connect the prediction with further evidence instantly | "It would be nice to have some more hints of a concrete nature. [...] For example, what were the most important indicators on the way to how the system now arrives at this? That you can look at it again, like in a timeline: Look, he's already had this event and he's just scrolling through it again. So that you can practically look it up again exactly by perhaps also looking at a timeline or at the criteria that were used. In the future itʼs probably also, I donʼt know, thereʼs a cross-link to interesting literature or: look, there were now 200 cases that are similarly published. You can then read them again. Here is the guideline and here are the therapy recommendations. Yes? […] That would be the next step, to think one step further" |
| Schwartz^15^ | interview | clinicians | Post-implementation | Performance (model) | Clinicians describe their trust as being influenced by their perceptions of the accuracy and correctness of CONCERN | "The more accurate it is, in my opinion...the more trust I have in the tool. |
| Schwartz^15^ | interview | clinicians | Post-implementation | Explanation needs | Clinicians’ ability to understand CONCERN was also confirmed to be an important factor influencing trust. Clinicians described wanting to evaluate the factors contributing to CONCERN to determine whether they trusted the prediction | "The CONCERN score has changed, like, you know, they’re now a yellow or whatever, it might be a good point to be like, oh, what do we think is contributing to that or even reviewing like, because I think there’s a way to review, like what, what went into that. And just being like, do we trust this? Do we not?" |
| Schwartz^15^ | interview | clinicians | Post-implementation | Knowledge base / Performance (model) | When CONCERN’s predictions aligned with clinicians’ impressions of the patient, their trust in the system was positively affected. a lack of concordance between CONCERN’s predictions and their assessments decreased their trust | "I felt good that it, very much aligned with how the patient was progressing, whether they were doing well or not doing so well"; "I think probably we all kind of take notice of it, but we don’t really talk about it because sometimes it doesn’t really correlate truly with how a patient is doing clinically" "That trust could be hindered, say, if I had a patient I was concerned about, and they were a green." |
| Schwartz^15^ | interview | clinicians | Post-implementation | Perceived usefulness (easier to complete task) | If there was concordance between their concern for a patient and the CONCERN prediction indicating high risk, they could use the prediction as evidence to support escalating care | "So, I guess in an instance, I could say that, like, oh, this patient’s CONCERN score is...red, like, this is just evidence that we need to do intervention" |
| Schwartz^15^ | interview | clinicians | Post-implementation | Perceived usefulness (easier to complete task) | For some clinicians, discordance between the CONCERN prediction and their impression of the patient did not diminish their trust as they viewed CONCERN as just 1 data point that they considered. | "There have been moments where I’m like, “oh, I’ve been in there all day, why is it not red?” But again, it’s not frequent enough for me to say, “oh, this is garbage.” I, I still respect its input [laughs]...it’s something I look at at the start of my shift...as the day goes on, I am taking note if there is a change." |
| Schwartz^15^ | interview | clinicians | Post-implementation | Perceived usefulness (easier to complete task) | Appreciating the prompt to pay attention to a patient and did not see any harm in an inaccurate prediction | "The one time it was off, I think, was just there was a lot of documentation happening for other reasons that weren’t a good clinical deterioration. There was just a lot of other things going on with this patient that required frequent documentation. And so, it was like a yellow. But again, it was nice to just know, like, oh, I should actually kind of see what’s been going on." |
| Schwartz^15^ | interview | clinicians | Post-implementation | Data quality | Others wanted to further scrutinize the rigor of model development | "I’d want to know a bit more about how it was developed, and so let’s say the data that CONCERN was trained on was exclusively ICU sepsis and organ failure, mortality, all-cause mortality, let’s say...then I would say this tool is only generalizable to the ICU setting, for example." |
| Schwartz^15^ | interview | clinicians | Post-implementation | Development | Some participants wanted to know that clinicians had been involved in the development of the system or that clinicians would have the opportunity to provide feedback on system performance after its implementation. | "I think just with anything, having someone who’s actually been there done that is way more, makes it, makes whatever you’re developing way more accurate, way more useful, way more diligent" |
| Schwartz^15^ | interview | clinicians | Post-implementation | Performance (model) | Clinicians expressed skepticism of the system’s ability to account for unique or complex patient characteristics | Because it’s so complicated, it’s quite, I mean, I hesitate to say unique, but there are a lot of a lot of factors in place. And it would be hard for a training data set to include enough patients who were similarly complex for it to have, let’s say, three hundred patients with infection in the right rib and the left shoulder and the left knee all at once. And for it [the system] to kind of know what to do at that point" |
| Schwartz^15^ | interview | clinicians | Post-implementation | Data quality | Clinicians described scenarios in which data that the system uses would be missing. Examples included emergencies, rapid deterioration, new patients, or when clinicians are burdened by work or documentation | "I’m not saying that systems like this aren’t smart, but I just feel like so much of it depends on what’s going on in that moment. And a lot of times, you know, our documentation isn’t always like right up to date with what’s going on at the moment" |
| Schwartz^15^ | interview | clinicians | Post-implementation | Perceived usefulness (easier to complete task) | Many clinicians mentioned that they would probably rely more on predictive CDSSs during the night shift, when they are assigned to care for more patients | "I’m like doing night coverage, so I don’t know the patients as well, so maybe I would, in that setting, be more reliant on a tool like that. "I’d say [CDSSs would be better than a clinician when] someone, a novice in their role. Like July [laughing about when new residents begin] or any new nurse or anything like that" |
| Schwartz^15^ | interview | clinicians | Post-implementation | Perceived usefulness (easier to complete task) | Clinicians described predictive CDSSs as better equipped to make predictions in situations where the change to the patient’s state is gradual rather than rapid | "Maybe the algorithm’s better at like kind of like nudging us to just like readdress some things that maybe are changing minutely day to day, so we may miss if we’re if we’re not, like, really aware of the trend" |
| Schwartz^15^ | interview | clinicians | Post-implementation | Explanation needs | Clinicians wanted global explanations, meaning information on how the CONCERN model calculates predictions generally | "So my questions are like, well, what kinds of phrases and words and how often, you know, is the is the CONCERN tool looking back? Is it, are they looking at one note? Are they looking at three notes? And when you say vital sign frequency, what does that mean?" "I think [to understand an algorithm like CONCERN] just more what it takes into account, whether it’s you know, their vital signs or their lab values, I don’t really know how it calculates, if they’re flagged as yellow or green." |
| Schwartz^15^ | interview | clinicians | Post-implementation | Explanation needs | Clinicians also wanted explanations for individual patient predictions provided at the point of care. | They wanted to see “the vital signs or the whatever that is making the score change” and “what piece of it is causing the algorithm to say that the person’s not stable” |
| Schwartz^15^ | interview | clinicians (some) | Post-implementation | Explanation needs | Some clinicians stated they did not need detailed explanations of CONCERN as it was just 1 component of their assessments | "The fact that it’s, it’s an extra data point that’s available to me there doesn’t make me so concerned about, well, you know, how does the machine learning work and when what goes into this? To me, I’m like, well, I understand what machine learning is and I understand that it helps me better inform some of my clinical decisions and maybe gives me like an extra reason to, to double check my, my own clinical assessment. So, in that sense, like I feel like it’s been a sufficient enough information for me" |
| Schwartz^15^ | interview | clinicians (some) | Post-implementation | Education and training | Some clinicians previously participated in CONCERN design focus groups, which they described as a helpful form of training, whereas others received formal training. Clinicians felt that formal training should be part of onboarding new staff. | “I guess like actually like having somebody go around and explain it… like, you know, the nurse educator, like how she comes around and she updates us on, like, the monitors and whatever, the educator. So she, maybe having someone come by and just be like, ‘hey, did you guys know there's this CONCERN score?’” |
| Schwartz^15^ | interview | clinicians (some) | Post-implementation | Education and training | Some clinicians had a poor understanding of CONCERN, with a few who felt that they did not receive education expressing frustration about this | “I would say that I wasn't given much training as to how I should be using it. And so I guess I keep an eye out for it and I take some time to look through it, understand what it means. But I would say that I don't yet like fully use or think that I can use it to its full potential.” |
| Schwartz^15^ | interview | clinicians | Post-implementation | Data quality | Knowing that their documentation would inform the CONCERN prediction, some nurses wanted to know that they were not missing something that would make the CONCERN prediction more accurate. Some said that they had or would change their documentation behaviour in attempts to make the prediction reflect their impression of the patient | I feel like I do try to put stuff in that’s like part of the CONCERN score, but it doesn’t always, like the CONCERN score doesn’t always reflect it, so then I’m like, I’m not sure I’m putting in the data correctly? Or like I’m just not putting it in the right comment boxes or like filling out my notes, you know, I don’t know if, like, I’m the one who’s not raising that level of concern because I’m just not putting, I’m not, like, doing the algorithm correctly where it would recognize it as a concern |
| Schwartz^15^ | interview | clinicians | Post-implementation | Evidence | Scientific evidence of the impact of a predictive CDSS on patient care was important for facilitating trust | I think, really like a study showing that the score has been used and the evidence behind it...if it’s published and peer reviewed, I think I definitely, personally I’d be more more inclined to use it. |
| Schwartz^15^ | interview | clinicians | Post-implementation | Evidence | Clinicians also described the importance of anecdotal reports of positive impact for the predictive CDSS | "I think anecdotally, like if others I know said, “hey, you know, I happened to catch this patient who was deteriorating and we were actually able to like, you know, get involved early and we were able to prevent this patient from either a rapid or like likely ICU transfer.” I think those things pull like a lot of weight." |
| Schwartz^15^ | interview | clinicians | Post-implementation | Actionability | Some clinicians wanted a clear recommendation for an action to take to trust the predictive CDSS | "Understanding how the predicting part comes in, I think would give me more confidence...some sort of like if/then tool, so if the score is greater than this, then you should take this kind of action" |
| Schwartz^15^ | interview | clinicians | Post-implementation | Performance (model)/ Evidence | A clinician expressed the importance of the predictive model being equitable | The one caveat [to machine learning] is it could, if it uses, you don’t know exactly what data it uses, and I would be interested in studies that explore whether that machine is systemically racist or classist or whatever...So, some sort of study to make sure it’s equitable to all populations is important. |
| Shulha^16^ | Feedback (informal interview and emails) | 2 Physicians | Low fidelity prototype | Design/  Actionability | Presenting the prediction in the most significant quadrant of the screen to ensure potential actionability violated the basic approach for clinical assessment and decision-making |  |
| Shulha^16^ | Feedback (informal interview and emails) | 2 Physicians | Low fidelity prototype | Explanation needs | Additional information to provide patient content would be relevant and would need to be incorporated into their assessment of the quality of the ML prediction. |  |
| Shulha^16^ | Feedback (informal interview and emails) | 2 Physicians | Low fidelity prototype | Explanation needs | Unclear how the consolidation in the image contributed to the severity score and how the severity score contributed to the risk of ICU admission |  |
| Shulha^16^ | focus groups with scenarios | Physicians | high fidelity prototype | Perceived usefulness (decreases workload) | Tool could be very useful in terms of planning for potential ICU admissions over time and helping manage staff resource issues and as an additional teaching resource with junior staff or nonradiology specialists. |  |
| Shulha^16^ | focus groups with scenarios | Physicians | high fidelity prototype | Perceived usefulness (easier to complete task) | The tool could be useful as an additional data source to confirm an assessment of low risk |  |
| Shulha^16^ | focus groups with scenarios | Physicians | high fidelity prototype | Patient / carer impact | Physicians noted the tool could be useful in explaining escalations in care to patients currently experiencing moderate symptoms |  |
| Shulha^16^ | focus groups with scenarios | Physicians | high fidelity prototype | Performance (model) | Not all physicians agreed with the tool’s assessments, but felt that more exposure to a larger number of predictions would be necessary for them to gauge how much they trusted the tool. |  |
| Zilker^17^ | Demonstration and interview | clinicians | high fidelity prototype | Understanding the explanation | Prefer suggested plots over common SHAP plots | “clearer and [are] easier to follow” They feel that SHAP plots are “confusing and not really self-explaining” because there are “toomany dots in the plot” |
| Zilker^17^ | Demonstration and interview | clinicians | high fidelity prototype | Perceived usefulness (easier to complete task) | Dashboard is helpful as a support for decision-making | “it is similar in processes to [their] own decision-making” (I1) and it is “prepared very well”  “believe these tools will not miss findings and will guide you to look at everything again when it automatically shows up in an overview”. |
| Zilker^17^ | Demonstration and interview | clinicians | high fidelity prototype | Perceived usefulness (easier to complete task) | All interviewees confirmed that interpretations in the form of the system’s dashboard would positively influence their trust in the predictions and it increases the acceptance of such predictions | because the dashboard provides “exactly what [they] work out [themselves] during a [...] visit”. Therefore, they think that it “increases [the] acceptance of such predictions” |
| Zilker^17^ | Demonstration and interview | clinicians | high fidelity prototype | Perceived usefulness (easier to complete task) (decreases workload) | Using predictive approaches is helpful | “Important for quick decision-making and provision or estimation of the required resources” “better assessment of patient risk, prioritization, [...], and transfer to another hospital if necessary” it fosters to “properly assess the patients” and to “order closer monitoring of the blood values”  it enables to “better assess capacity”, so that practitioners “can also adjust ICU beds” |
| Zilker^17^ | Demonstration and interview | clinicians | high fidelity prototype | Perceived usefulness (easier to complete task) (decreases workload) | The admission to ICU is helpful for medical experts with less experience | “For younger colleagues [...] who don’t have that much experience with [determination of ICU admission] yet, it’s certainly helpful, also just to make sure that nothing is overlooked” |
| Zilker^17^ | Demonstration and interview | clinicians | high fidelity prototype | Explanation needs | Understanding the prediction is necessary for trust | “would not trust such predictions without further explanation” as they “think it’s important to know what the decision is based on” Clinicians “have to justify the process to the patients and relatives and to do that [they] want to understand the process to be able to justify it” |

## Table D. Data from extraction and analysis – design and usability

| **Author** | **Method** | **Participant** | **Stage of development** | **Explanation method** | **Specific features** | **Design category** | **Result** | **Example quote (if available)** |
| --- | --- | --- | --- | --- | --- | --- | --- | --- |
| Abraham^2^ | Cognitive walkthrough and interview | All | Prototype | SHAP | Preferred risk as a percentage > qualitative categories (low/med/high) | Numeric information | Most clinicians preferred percentage-risk (n = 8) estimates over qualitative risk categories (low/medium/high, n = 3), calling percentages “more meaningful (C4, CRNA).” No difference in preferences across clinician roles was noted. | “I like seeing the percentage because it would enable me to compare this patient to other patients more easily. And I think that most people would have a sense about whether 13 % chance of AKI– especially if they get these [tool] reports for many patients– they’ll have a sense of if that’s a low or a high number (C5, Anesthesiologist).” |
| Abraham^2^ | Cognitive walkthrough and interview | All | Prototype | SHAP | Table and barchart | Charts and graphs | the table (Fig. 1a, n = 10) and bar chart (Fig. 1b, n = 7) were the preferred complication risk visualizations. | “Tables, grids or graphs, in general, are better [than paragraphs] (C1, Anesthesiologist).” |
| Abraham^2^ | Cognitive walkthrough and interview | All | Prototype | SHAP | Narrative text explanation | Layout of information | Most clinicians found it hard to extract information quickly from the narrative presentation format (Fig. 2c) and felt the format failed to direct their attention to important details. | “Tables, grids or graphs, in general, are better [than paragraphs] (C1, Anesthesiologist).” |
| Abraham^2^ | Cognitive walkthrough and interview | All | Prototype | SHAP | bar graph color coding | Colour coding information | Respondents felt the bar graph’s visually distinctive bars and color-coding “draws [sic] your eye to what stands out (C4, CRNA).” | “I like red, yellow, and green in terms of things that need to catch my attention. Yellow, like meh. Green, don’t worry about it (C2, Anesthesiologist).” |
| Abraham^2^ | Cognitive walkthrough and interview | All | Prototype | SHAP | Color coding | Colour coding information | Many clinicians endorsed highlighting elevated risks in red to establish familiar colorcoding with risk urgency (n = 8), and many also felt that risks should be ranked from most to least likely (n = 14) for easy identification of the highest priority concerns. | “rank color cod(ing) by severity,” and suggested, “keeping delirium red, and then [using] a different color for second [highest risk] so it’s… red, yellow, green, depending on where they fall in the threshold (C17, Anesthesiologist).” |
| Abraham^2^ | Cognitive walkthrough and interview | All | Prototype | SHAP | Ordering features | Display of features | Clinicians preferred risk factors in descending order of added risk, as in Fig. 2c (n = 10; 6 anesthesiologists, 3 CRNAs, and 1 ICU RN). Many anesthesiologists and CRNAs stated that this helped them quickly find sources of increased risk by spatially separating them from protective factors. | “I like the one like you have on the right where you look at the protective and the risk factors and you see where the biggest contributions are at the top here (C1, Anesthesiologist).” Another agreed, adding that “I think grouping it according to those that increase, and those that decrease, at least from my standpoint, the way my mind works, I think, is, would be the cleanest and most logical (C12, Anesthesiologist).” |
| Abraham^2^ | Cognitive walkthrough and interview | All | Prototype | SHAP | Categorising risk factors | Display of features | Organising risk factors preoperatively and intraoperatively (Fig. 2d) made modifiable (intraoperative) risk factors easier to find (n = 6; 3 surgeons, 1 CRNAs, 1 anesthesiologist). Placing earlier and later measurements in separate panels helped them make more sense of each factor since some factors could not be present based solely on preoperative data. | “I think having preoperative and intraoperative is interesting and potentially helpful. Because [the tool user]… might say, ‘what amount of this patient’s risk was produced by how they came to the OR? Then, what amount was produced by how they behaved during the surgery?’ (C1, Anesthesiologist).” |
| Anjara^3^ | Think aloud | Oncologists | Prototype | example-based explanations | Order of patient attributes contributing to score | Display of features | Participants highlighted the lack of order of priority in patient attributes displayed in the explanation. The important attributes contributing to the relapse score was not displayed in an intuitive way. | “The descriptive part of the different variables are confusing. Because obviously there are some that are really important, but others could be too general. For example, the comorbidity, cardiopathy is just like saying nothing because it could be [a] very important issue or it could be arrythmia, well controlled, with no implications. In the same category, dyslipidemia could mean nothing in the evolution of the patient. It depends. Hypertension could be very difficult to control. I think it is quite general. Also, a smoker, how much? I don’t know, it is too much information and too general to modify my individual decision over a patient.”– |
| Barda^4^ | guided review/ questionnaire | all | low fidelity prototype | SHAP |  | Numeric information | Expressing risk as percent probability (reduced information processing) | “I actually took out a calculator and based on the odds, I calculated the patient’s percent probability of death.”  “For me, percent risk of mortality is going to be easier to interpret than the odds.” |
| Barda^4^ | guided review/ questionnaire | all | low fidelity prototype | SHAP |  | Display of features | Aggregating information (e.g., grouping all features related to a measurement) (reduced information processing) | “I really appreciate the groupings on this graph…I said well there’s only an 8% risk of death but it’s being driven largely by this neuro bucket, so what’s going on there?” |
| Barda^4^ | guided review/ questionnaire | nurses | low fidelity prototype | SHAP |  | Layout of information | Minimal, actionable information and tended to prefer simpler, static explanations organized by assessment groups |  |
| Barda^4^ | guided review/ questionnaire | physicians | low fidelity prototype | SHAP |  | Layout of information | More dynamic explanations. |  |
| Barda^4^ | guided review | all | low fidelity prototype | SHAP |  | Layout of information | High-level information with details available on demand |  |
| Barda^4^ | guided review | all | low fidelity prototype | SHAP |  | Charts and graphs | Interactive options to support different displays/ organizations for various users | “I also like that clicking on the graph directs you to the associated lab/physical assessment in the data table.” “Is it possible that when you click on lactate results it could both bring up the raw data graph as well as the little components of the lactate table? |
| Barda^4^ | guided review | all | low fidelity prototype | SHAP | Table of raw feature values | Display of features | Helpful when examining trend-based features and interpreting the discretized features used in the explanation. The exact amount of the increase, the current value, and the minimum value were all described as helpful information |  |
| Barda^4^ | guided review | all | low fidelity prototype | SHAP | Table of raw feature values | Display of features | Directionality for trend-based features | "It would be nice if I could just say, 'pulse ox is decreasing, creatinine is increasing'" |
| Barda^4^ | guided review | all | low fidelity prototype | SHAP | Table of raw feature values | Layout of information | Simpler terminology | Simpler terminology for trend-based features in the explanation |
| Barda^4^ | guided review | all | low fidelity prototype | SHAP | Time-series data plot | Charts and graphs | Multiple plots | Multiple plots to view and compare data |
| Barda^4^ | guided review | all | low fidelity prototype | SHAP | Time-series data plot | Charts and graphs | Highlight points related to features | Highlighting the specific points used to derive features in the model |
| Barda^4^ | guided review | all | low fidelity prototype | SHAP | Time-series data plot/ table of raw features | Charts and graphs | Auto-population of data | Auto-populating data on these plots when an element was selected in the explanation plot or table of raw features |
| Barda^4^ | guided review | all | low fidelity prototype | SHAP | Time-series data plot | Charts and graphs | Investigate suspicious values (e.g., outliers or errors), assess trend-based features and determine patient baselines) |  |
| Barda^4^ | guided review | all | low fidelity prototype | SHAP | Contextual information | Context | Providing clinical context information | Although the prototypes contained only minimal contextual patient information, those details were considered to be important in assessing the clinical relevance of a prediction. Interventions were seen as particularly important. For example, a high-risk prediction driven by a low Glasgow Coma Score measurement would be of less concern to a provider if they knew the patient was sedated and paralyzed at the time of the measurement. |
| Barda^4^ | guided review | all | low fidelity prototype | SHAP | Contextual information | Context | Prominent display of baseline risk | Informants also noted that baseline risk levels provided important context when interpreting predictions (e.g., a predicted risk of 40% was more concerning if they knew the baseline risk was 2%), and suggested moving this information to a more prominent display location. |
| Barda^4^ | guided review | all | low fidelity prototype | SHAP | Contextual information | Charts and graphs | Inclusion of risk trends | including risk trends would improve model utility, as a change in risk might be more clinically relevant than a single predicted risk (e.g., a patient that has had a high predicted risk for several days might be of less concern than a patient with a lower predicted risk that recently increased). |
| Barda^4^ | guided review/ questionnaire | all | low fidelity prototype | SHAP | Tornado plot > force plot | Charts and graphs | Some providers had found that the force plot was confusing to interpret |  |
| Besculides^5^ | Interviews | Dieticians | Implemented | SHAP | Hover tool displays top features | Layout of information | Aware of hover tool (although not all used it) and understood that the factors listed in it were those that contributed the most to the score. | All RDs had knowledge of the interface’s hover tool (although not all used it) and understood that the factors listed in it were those that contributed the most to the score. ; “I wish I knew exactly how it worked or how the algorithm captures these patients.” |
| Besculides^5^ | Interviews | Dieticians | Implemented | SHAP | Hover tool displays top features | Display of features | Misunderstood how features were displayed | Some thought the factors were always the same in different orders, but others knew they changed based on the patient. |
| Bienefeld^6^ | Interviews | Clinicians | Prototype (high fidelity) testing | SHAP | Shapley values of contributers in heatmap | Charts and graphs | Shapley values in the heatmap are NOT helpful to increasing their understanding and trust in the system (too complex) | “Here [referring to Fig. 2B 1], is the overall risk score of 0.8 and here [referring to Fig. 2C 2], I see what contributes to the baseline risk. That makes sense. But this information over here [referring to Shapley values of dynamic contributors, Fig. 2E], I don’t need to know all this, I am not a mathematician. What I need to know is do these results [referring to Fig. 2B 1] make sense clinically […]. For instance, when I see that on November 30th [Fig. 2E 3], the risk for DCI was high, then I want to know what we did that day; like did we do a CT scan, and did [the patient] actually develop a DCI? Also, if I administered Mannitol [medication to lower intracranial pressure17], would the risk go down then? These kinds of things I need to know to trust [the DCIP].” |
| Fritz^7^ | focus groups | physician and nurses | possible display elements | SHAP |  | Layout of information | making additional details available either by hovering or by clicking to a new screen might be an effective strategy to prevent information overload. | “So having the most relevant information on screen, and then either hovering or clicking on a piece of information to get things like confidence bounds, more details that are not useful immediately but are more useful for understanding.” |
| Fritz^7^ | focus groups | physician and nurses | possible display elements | SHAP | Color | Colour coding information | using red color to indicate high risk would target the user to that information first. | “We’re all so color sensitive. … So like for me, by including red stuff on there, my brain just knows, I keep thinking I need to look at that first.” |
| Fritz^7^ | focus groups | physician and nurses | possible display elements | SHAP | graphs | Charts and graphs | View trends over time in graphical format | “For some reason, I look at the graph and I can assimilate that information quicker, and it requires less brain cells to digest.” |
| Fritz^7^ | interviews | physician and nurses | low fidelity prototype | SHAP |  | Layout of information | labels and text descriptions were inadequate in a few places within the prototype display interface | “If I weren’t being guided, I wouldn’t necessarily know that OR room is the place to click. Maybe if there was a box around it or something that looked like a button, that may be a little more intuitive.” Participant 1 (CRNA) “It would be useful to know what the definition [of the complication] is, just briefly.” Participant 4 (attending) |
| Fritz^7^ | interviews | physician and nurses | high fidelity prototype | SHAP | color | Colour coding information | Color coding to indicate risk was confusing as thresholds differed across surgical services | Multiple participants initially found it confusing that one patient’s risk prediction might be colored green when another patient had a numerically lower risk that was colored red. “I expected the red to be on top, but it’s the higher value that’s on top. So we have—I think that needs a little differentiation for me. So the color provides one piece of information and the numeric provides the other?” - Participant 1 (CRNA) |
| Fritz^7^ | interviews | physician and nurses | high fidelity prototype | SHAP | Displaying as percentage | Numeric information | Participants did not notice that the percentages could take negative values, indicating the variable was associated with reduced risk of the complication rather than increased risk. | “I’m a little confused why some of these are negative, but it seems like it has a strong contribution.” |
| Fritz^7^ | interviews | physician and nurses | high fidelity prototype | SHAP |  | Numeric information | Would prefer contributions were scaled to 100% | Other participants were confused why the percentages did not add up to 100%, because the Shapley values were scaled such that they added up to the patient’s predicted risk rather than to 100%.  “Why do these sum to more than 100%? Is it like a relative scaling thing?” |
| Fritz^7^ | interviews | physician and nurses | high fidelity prototype | SHAP |  | Display of features | Participants wanted clearer labels for the levels of categorical variables | “But I do think, for your categorical variables, it’d be nice if you could re-map them to the actual category to be a little easier to interpret.” |
| Fritz^7^ | interviews | physician and nurses | high fidelity prototype | SHAP |  | Numeric information | Participants wanted to reduce the number of significant figures displayed for numeric variables |  |
| Fritz^7^ | interviews | physician and nurses | high fidelity prototype | SHAP |  | Display of features | Want to see classes of features rather than just individual features | “One thing we might consider is…some sort of basic mechanism, or some sort of summary mechanism to say these are non-modifiable preoperative things that determine the patient’s risk.” |
| Fritz^7^ | interviews | physician and nurses | high fidelity prototype | SHAP |  | Display of features | Want to omit features making very small contributions | “Let’s say there was a less than 1% [contribution]. And then I could click it here and it would show me all the other stuff that’s in the model. I think I’d be able to better focus on these things.” |
| Fritz^7^ | interviews | physician and nurses | high fidelity prototype | SHAP |  |  | unclear which prediction is newest versus oldest | “Okay. And then these data points here, are these I guess baseline, like initial?” |
| Fritz^7^ | interviews | physician and nurses | high fidelity prototype | SHAP |  | Display of features | unclear which features are in the model | “I didn’t appreciate it right away, so I did not know.” |
| Fritz^7^ | interviews | physician and nurses | high fidelity prototype | SHAP | color | Colour coding information | want features color coded | “Would it be helpful to have negative contributing factors in red or something like that?” |
| Fritz^7^ | interviews | physician and nurses | high fidelity prototype | SHAP |  | Display of features | unclear abbreviations for features | “What is this PPM? Is it permanent pacemaker?” |
| Helman^8^ | focus group | all | Prototype | Feature importance |  | Charts and graphs | Participants struggled to understand arrow directionally and their meaning regarding the listed clinical indicators (fluid responsiveness, arterial tone, and cardiac performance). |  |
| Helman^8^ | focus group | all | Prototype | Feature importance |  | Layout of information | simplicity of design | The initial GUI prototype was noted to be visually busy. |
| Jauk^10^ | group meeting | all | implemented | Feature importance | color | Colour coding information | appreciated that high risk patients were presented with a yellow symbol and very high risk patients with a red symbol | “I like the presentation with the traffic light symbol.” |
| Jauk^10^ | group meeting | all | implemented | Feature importance |  | Numeric information | the risk of delirium had been visualized using percentages. This was criticized by the experts, as their interpretation was not clear to them. |  |
| Jauk^10^ | group meeting | all | implemented | Feature importance |  | Charts and graphs | the percentages were replaced by a bar chart visualizing the three risk categories and an arrow indicating the location of a patient on the risk dimension. | “The bar representing the range of delirium risk helps us to identify patients at the border to another risk group.” |
| Jin^11^ | case study | cardiovascular doctor | high fidelity prototype 2 | Similar patients | similar patient view (the aggregated form): "click to highlight function" corresponding disease progression paths automatically highlighted by the system | Colour coding information | Automatically highlighting the similar patients and their corresponding disease progression paths was "very informative" | “clearly show the progression of an outcome in context of treatments.” He felt that this view gave him more confidence in the prediction results as “it provides specific evidence [to support the prediction results].” |
| Jin^11^ | interview | physicians | high fidelity prototype 2 | feature importance | outcome analysis task | Context | revealing the impact of historical event on the prediction result is useful. | "We sometimes hesitate to trust the machine learning models because they usually fail in providing reasons. It can help raise our confidence if the system can illustrate how the model [arrives] at the result.” |
| Jin^11^ | interview | physicians | high fidelity prototype 2 |  |  | Charts and graphs | these new complex views (designs) were more informative when compared to more familiar statistical charts |  |
| Jin^11^ | interview | physicians | high fidelity prototype 2 |  |  | Layout of information | operating multiple coordinated views also takes some effort | that it would easier to use if the tool could directly generate and print out a report without as many interactions.  “It will be easier to use if you could somehow separate the views of three different functions apart into multiple pages and guide the operation in a step to step manner instead of packing them all together.” |
| Jin^11^ | interview | physicians | high fidelity prototype 2 |  |  | Context | that providing the information of historical medical records is not enough | Although historical medical records are important, doctors also want to input the vital signs of the patients into the system.” |
| Jin^11^ | interview | physicians | high fidelity prototype 2 |  |  | Context | Insufficient context information: the information shown in the description view was helpful, but they would like to have more. | They suggested we collect more information, such as the latest diagnosis guidelines and information about new medicines. |
| Matthiesen^12^ | walkthrough | cardiologists | near-live | LIME | Probability as percentage with decimals | Numeric information | Showing the probability score as a percentage with decimals created uncertainty | "Yes, I think again that if you present 58.6% then it expresses an accuracy that you may have difficulty navigating with. I know it from other areas in the medical world, the thing about expressing something with a decimal number, it expresses an accuracy for which there may be no evidence at all […] I have a hard time relating to the number […] it’s problematic to translate that into something clinically relevant." |
| Matthiesen^12^ | walkthrough | cardiologists | near-live | LIME |  | Layout of information | The naming of the parameters were sometimes found difficult to interpret |  |
| Matthiesen^12^ | walkthrough | cardiologists | near-live | LIME |  | Display of features | The probability score and the presentation of important parameters can reduce information search time, because the tool summarizes the valuable information in a table | Typically, electrophysiologists must retrieve valuable information by clicking through multiple webpages in the ICD manufacturer’s web-based system, which the prediction tool summarizes in a table |
| Matthiesen^12^ | interview | cardiologists | near-live | LIME |  | Layout of information | Positive or negative predictive value should be as unambiguous as possible, showing either low or high risk when the alarm is raised | "If you want to come out with this, it must be something with a positive predictive value that is really good, so that you don’t get a lot of nonsense that you can’t use. The alarm should only be raised when there really is something" |
| Pinto^13^ | interview | data scientists |  |  | time plots | Charts and graphs | Wanted to visualize the time plots of some features | Many wanted to visualize the time plots of some features, particularly spectral bands' relative powers. Whenever possible, we advise the use of spectral-band features. |
| Shulha^16^ | focus groups | Physicians | low fidelity prototype | LIME |  | Charts and graphs | Display of multiple images in comparison |  |
| Shulha^16^ | focus groups | Physicians | low fidelity prototype | LIME | toggle | Charts and graphs | Turn the heat map overlay on and off so as to be able to compare the areas highlighted by the ML model with the actually affected areas |  |
| Shulha^16^ | focus groups with scenarios | Physicians | high fidelity prototype | LIME |  | Context (barrier) | One of the most important issues uncovered was the degree to which physicians erroneously assumed that the additional data present in the tool, namely vitals and laboratory values, were being included in the x-ray severity score |  |
| Shulha^16^ | focus groups with scenarios | Physicians | high fidelity prototype | LIME | toggle | Charts and graphs | Appreciated the ability to toggle the explainability overlay so that both options made it easier for them to assess how consistently the tool was identifying elements of the x-ray image they felt would contribute to overall disease severity |  |
| Zilker^17^ | Demonstration and interview | clinicians | high fidelity prototype | SHAP and GAMs | Interpretation plots (provides further interpretation details on selected static or sequential medical indicators.) | Charts and graphs | All interviews confirmed the usefulness of the interpretation plots to understand at a glance what caused the prediction | “find[s] it helpful in any case [to] have an overview of which factors have been included [and to] [...] understand at a glance what caused the system to do this. [...] Because then [they] can also take a closer look at what the blood pressure is doing or where the leukocytes are”. |
| Zilker^17^ | Demonstration and interview | clinicians | high fidelity prototype | SHAP and GAMs | Visual plots | Charts and graphs | Visual plots are the language that is spoken medically |  |
| Zilker^17^ | Demonstration and interview | clinicians | high fidelity prototype | SHAP and GAMs | Interpretation plots (provides further interpretation details on selected static or sequential medical indicators.) | Charts and graphs | Prefer simple plots because they usually have to act relatively quickly | “there are always so many new colleagues [...] in the clinic that I think keeping it as simple as possible makes the most sense” (I2) and that they usually have to act “relatively quickly” |
| Zilker^17^ | Demonstration and interview | clinicians | high fidelity prototype | SHAP and GAMs |  | Layout of information | Providing interpretations in the form of "written text" |  |

## References

1. Critical Appraisal Skills Programme. CASP Qualitative Studies Checklist. 2023. <https://casp-uk.net/casp-tools-checklists/qualitative-studies-checklist/>.
2. Abraham J, Bartek B, Meng A, et al. Integrating machine learning predictions for perioperative risk management: towards an empirical design of a flexible-standardized risk assessment tool. *Journal of biomedical informatics* 2023; **137**: 104270.
3. Anjara SG, Janik A, Dunford-Stenger A, et al. Examining explainable clinical decision support systems with think aloud protocols. *PLoS ONE* 2023; **18**(9 September): e0291443.
4. Barda AJ, Horvat CM, Hochheiser H. A qualitative research framework for the design of user-centered displays of explanations for machine learning model predictions in healthcare. *BMC Med Inform Decis Mak* 2020; **20**(1): 257.
5. Besculides M, Mazumdar M, Phlegar S, et al. Implementing a Machine Learning Screening Tool for Malnutrition: Insights From Qualitative Research Applicable to Other Machine Learning-Based Clinical Decision Support Systems. *JMIR formative research* 2023; **7**: e42262.
6. Bienefeld N, Boss JM, Luthy R, et al. Solving the explainable AI conundrum by bridging clinicians' needs and developers' goals. *NPJ Dig Med* 2023; **6**(1): 94.
7. Fritz BA, Pugazenthi S, Budelier TP, et al. User-Centered Design of a Machine Learning Dashboard for Prediction of Postoperative Complications. *Anesth Analg* 2024; **138**(4): 804-13.
8. Helman S, Terry MA, Pellathy T, et al. Engaging Multidisciplinary Clinical Users in the Design of an Artificial Intelligence-Powered Graphical User Interface for Intensive Care Unit Instability Decision Support. *Appl Clin Inform* 2023; **14**(4): 789-802.
9. Henry KE, Kornfield R, Sridharan A, et al. Human–machine teaming is key to AI adoption: clinicians’ experiences with a deployed machine learning system. *NPJ digital medicine* 2022; **5**(1): 97.
10. Jauk S, Kramer D, Avian A, Berghold A, Leodolter W, Schulz S. Technology Acceptance of a Machine Learning Algorithm Predicting Delirium in a Clinical Setting: a Mixed-Methods Study. *JOURNAL OF MEDICAL SYSTEMS* 2021; **45**(4).
11. Jin Z, Cui S, Guo S, Gotz D, Sun J, Cao N. CarePre. *ACM Transactions on Computing for Healthcare* 2020; **1**(1): 3344258.
12. Matthiesen S, Diederichsen SZ, Hansen MKH, et al. Clinician Preimplementation Perspectives of a Decision-Support Tool for the Prediction of Cardiac Arrhythmia Based on Machine Learning: Near-Live Feasibility and Qualitative Study. *JMIR Hum Factors* 2021; **8**(4): e26964.
13. Pinto MF, Batista J, Leal A, et al. The goal of explaining black boxes in EEG seizure prediction is not to explain models' decisions. *Epilepsia Open* 2023; **8**(2): 285-97.
14. Samhammer D, Roller R, Hummel P, et al. "Nothing works without the doctor:" Physicians' perception of clinical decision-making and artificial intelligence. *Front Med* 2022; **9**(101648047): 1016366.
15. Schwartz JM, George M, Rossetti SC, et al. Factors Influencing Clinician Trust in Predictive Clinical Decision Support Systems for In-Hospital Deterioration: Qualitative Descriptive Study. *JMIR HUMAN FACTORS* 2022; **9**(2).
16. Shulha M, Hovdebo J, D'Souza V, Thibault F, Harmouche R. Integrating Explainable Machine Learning in Clinical Decision Support Systems: Study Involving a Modified Design Thinking Approach. *JMIR Form Res* 2024; **8**(101726394): e50475.
17. Zilker S, Weinzierl S, Kraus M, Zschech P, Matzner M. A machine learning framework for interpretable predictions in patient pathways: The case of predicting ICU admission for patients with symptoms of sepsis. *Health Care Manag Sci* 2024; (dl1, 9815649).

## Abbreviations

ACT: Anaesthesiology Control Tower

AI: Artificial intelligence

AI-DSS: Artificial Intelligence–driven Decision Support Systems

AKI: Acute Kidney Injury

ASA: American Society of Anaesthesiologists Physical Status Classification System

BMI: Body mass index

CDSS: Clinical decision support system

CKD: Chronic kidney disease

CONCERN: Communicating Narrative Concerns Entered by Registered Nurses

CRNA: Certified Registered Nurse Anaesthetist

DCI: Delayed Cerebral Ischemia

ED: Emergency Department

EHR: Electronic health record,

EEG: electroencephalogram,

GAM: Generalized Additive Models

GUI: Graphical User Interface

ICD: International Classification of Diseases

ICU: Intensive care unit,

IDSS: Intelligent Decision Support System

LIME: Local Interpretable Model-agnostic Explanations

MAP: Mean Arterial Pressure

ML: Machine learning,

MUST-Plus: Malnutrition Universal Screening Tool,

OR: Operating room

RD: Registered dietician

SHAP: SHapley Additive exPlanations,

VT/VF: Ventricular Tachycardia / Ventricular Fibrillation
